# Switching the O-O Bond Formation Pathways of Ru-pda Water Oxidation Catalyst by Third Coordination Sphere Engineering

**DOI:** 10.34133/2021/9851231

**Published:** 2021-04-13

**Authors:** Yingzheng Li, Shaoqi Zhan, Lianpeng Tong, Wenlong Li, Yilong Zhao, Ziqi Zhao, Chang Liu, Mårten S. G. Ahlquist, Fusheng Li, Licheng Sun

**Affiliations:** ^1^State Key Laboratory of Fine Chemicals, Institute of Artificial Photosynthesis, DUT-KTH Joint Education and Research Centre on Molecular Devices, Dalian University of Technology, Dalian 116024, China; ^2^Department of Theoretical Chemistry and Biology, School of Engineering Sciences in Chemistry Biotechnology and Health, KTH Royal Institute of Technology, 10691 Stockholm, Sweden; ^3^Department of Chemistry, University of California, Riverside, California 92521, USA; ^4^School of Chemistry and Chemical Engineering/Institute of Clean Energy and Materials, Guangzhou University, No. 230 Wai Huan Xi Road, Higher Education Mega Center, Guangzhou 510006, China; ^5^Department of Chemistry, School of Engineering Sciences in Chemistry, Biotechnology and Health, KTH Royal Institute of Technology, 10044 Stockholm, Sweden; ^6^Center of Artificial Photosynthesis for Solar Fuels, School of Science, Westlake University, 310024 Hangzhou, China

## Abstract

Water oxidation is a vital anodic reaction for renewable fuel generation via electrochemical- and photoelectrochemical-driven water splitting or CO_2_ reduction. Ruthenium complexes, such as Ru-bda family, have been shown as highly efficient water-oxidation catalysts (WOCs), particularly when they undergo a bimolecular O-O bond formation pathway. In this study, a novel Ru(pda)-type (pda^2–^ =1,10-phenanthroline-2,9-dicarboxylate) molecular WOC with 4-vinylpyridine axial ligands was immobilized on the glassy carbon electrode surface by electrochemical polymerization. Electrochemical kinetic studies revealed that this homocoupling polymer catalyzes water oxidation through a bimolecular radical coupling pathway, where interaction between two Ru(pda)–oxyl moieties (I2M) forms the O-O bond. The calculated barrier of the I2M pathway by density-functional theory (DFT) is significantly lower than the barrier of a water nucleophilic attack (WNA) pathway. By using this polymerization strategy, the Ru centers are brought closer in the distance, and the O-O bond formation pathway by the Ru (pda) catalyst is switched from WNA in a homogeneous molecular catalytic system to I2M in the polymerized film, providing some deep insights into the importance of third coordination sphere engineering of the water oxidation catalyst.

## 1. Introduction

In a typical solar fuel generation device of either electrochemical or photoelectrochemical driven, it often consists of a fuel-forming cathodic half-reaction, such as hydrogen evolution reaction (HER), carbon dioxide reduction reaction (CO_2_-RR), or nitrogen reduction reaction (NRR), and acquires protons and electrons. Water oxidation (2H_2_O → O_2_ + 4H^+^ + 4e^−^), on the other hand, plays the unique role of an anodic half-reaction that provides protons and electrons in the overall reaction of fuel generation [1, 2]. This oxidation process is a four-proton four-electron process, which is not only thermodynamically demanding but also kinetically sluggish. Therefore, developing highly efficient water oxidation catalysts to promote this process is imperative for any practical application of HER, CO_2_-RR, and NRR [3, 4]. To meet this demand, a comprehensive understanding of the catalytic mechanism of the water oxidation reaction is required [5, 6]. Studying a multielectron and multiproton transfer process is a difficult task where molecular catalysts with tailor-designed organic ligands show their advantages of precise structures and relatively easy identification of intermediates involved in the catalytic processes.

**Scheme 1:**
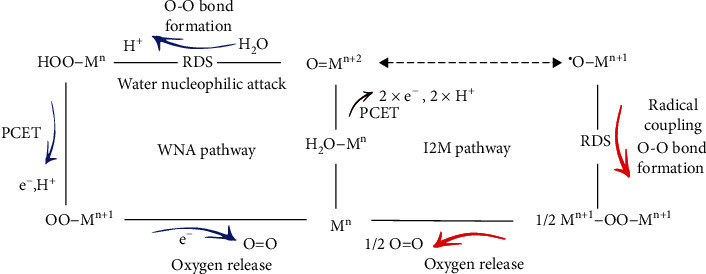
The overview of WNA and I2M O-O bond formation pathways for the water oxidation reaction.

Molecular water oxidation catalysts (MWOCs) containing various transition metal cores have therefore drawn significant attention in recent years, because their structures can be readily tailored for systematic structure-reactivity investigation, and their homogeneous catalytic property allows illustration of reaction mechanisms using classic spectroscopic techniques [7–9]. According to the mechanistic study upon numerous MWOCs, the O-O bond formation is often believed to be the rate-determining step (RDS) with the highest energy barrier through the catalytic water oxidation cycle [8, 10]. Two major approaches have been proposed and widely accepted for the essential O-O bond formation step (Scheme 1): the water nucleophilic attack mechanism (WNA) and the oxygen radical coupling mechanism (I2M). Briefly, proton-coupled redox reaction upon the metal center (M^n^) of a MWOC yields a high valence metal-oxo (M^n+2^=O) species, which can either be attacked by a water molecule in a nucleophilic manner, forming the O-O bond (WNA), or transform to a corresponding metal-oxyl radical (M^n+1^-O^•^) intermediate, immediately affording a peroxo-bond with another adjacent metal-oxyl radical (I2M) [11].

Among Ru-based MWOCs, the family of mononuclear Ru(bda)L_2_ (bda^2-^ = 2,2'-bipyridine-6,6'-dicarboxylic acid, L = N–cyclic aromatic ligands, e.g., pyridine) complexes have shown outstanding performances in terms of catalytic turnover frequency (TOF) and overpotential [12–14]. It has been demonstrated that water oxidation by these complexes proceeds through a dinuclear I2M pathway and the O-O bond forms via intermolecular coupling of two formal Ru^V^=O moieties with significant Ru^IV^-oxyl character. The hydrophobic nature of the Ru^V^=O species was believed to be one major force to drive the bimolecular radical coupling of Ru (bda) under aqueous catalytic conditions [15, 16]. The series of Ru(pda)L_2_ (pda^2-^ =1,10-phenanthroline-2,9-dicarboxylate) complexes have remarkably similar coordination geometry with the Ru(bda)L_2_ catalysts. However, small modification of the backbone organic ligands from bda to a relatively rigid pda dramatically altered the catalytic pathway of the O-O bond formation. Unlike Ru(bda)L_2_, Ru(pda)L_2_ prefers a WNA water oxidation pathway when using ceric ammonium nitrate as a sacrificial oxidant [17].

Density-functional theory (DFT) study displayed that, compared with the more flexible bda backbone ligand, the pda ligand increased the steric repulsion among catalysts and thus hindered two formal (pda)Ru^V^=O species from approaching each other [17]. Meanwhile, the (pda)Ru^V^=O species possesses less Ru^IV^-oxyl radical character than the (bda)Ru^V^=O species, and less likely to proceed through the I2M pathway [18]. For the case study of Ru(bda)L_2_ as well as its analogs illustrate that the catalytic behavior of Ru MWOCs can be affected by even a small change of the primary coordination environment [17]. While the orientation of hydrophobic/hydrophilic substituent groups of MWOCs is a key factor contributing to the different reaction mechanisms [18], modifying the third coordination sphere of the catalyst is another approach to adjust their local environment and thus facilitate the I2M O-O bond formation pathway. These findings encouraged us to manipulate the catalytic water oxidation pathway of Ru(pda) complexes using combined strategies of direct ligand modification and local catalytic environment design.

**Scheme 2:**
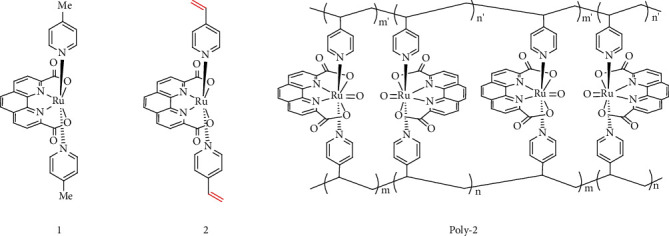
Molecular structures of 1, 2, and poly-2.

Herein, two Ru(pda) MWOCs were synthesized with 4-picoline (1) and 4-vinylpyridyl (2) as axial ligands, respectively (Scheme 2). Kinetic studies revealed that electrochemical-driven water oxidation by 1 went through a WNA O-O bond formation pathway, similarly to what was observed in the previous Ce^IV^-driven water oxidation by 1. Nevertheless, complex 2 after immobilized on the glassy carbon (GC) electrode surface by electropolymerization (this homocoupling polymer is denoted as poly-2) in triggers intermolecular O-O radical coupling between metal-oxyl radicals during the electrocatalytic water oxidation process. Our experimental results clearly revealed that the WNA O-O bond formation pathway of single-site MWOCs can switch to the I2M mechanism by changing the local environment, such as shortening the intermolecular distance of catalyst active center, via a homopolymerization method in this case.

## 2. Results and Discussion

**Figure 1:**
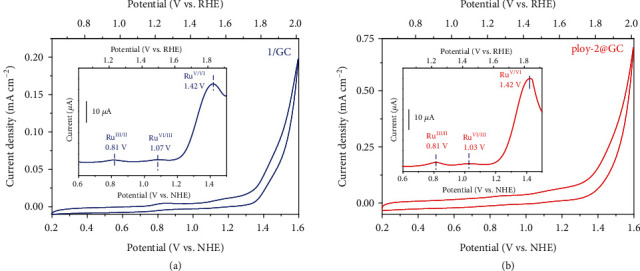
Cyclic voltammograms (CVs) of 1/GC (a) and poly-2@GC (b) measured in a pH = 7.0 sodium phosphate buffer (100 mM) at a scan rate of 100 mV s^−1^. Insets show the corresponding differential pulse voltammograms (DPVs).

Electrochemical properties of complex 1 in solution and complex 2 that is immobilized on glass carbon (GC) electrode via electropolymerization were investigated by cyclic voltammograms (CVs) and differential pulse voltammograms (DPVs) (as shown in Figure 1). The voltammograms were recorded in a neutral sodium phosphate buffer (pH = 7.0, ionic strength = 1 M) containing 10% (*v*/*v*) CH_3_CN and glassy carbon (0.071 cm^2^) electrodes were employed as the working electrodes for the measurement of complex 1 (1/GC) under homogeneous conditions and polymerized complex 2 (poly-2@GC) under heterogeneous conditions. The CV of poly-2@GC exhibited consecutive redox waves of Ru^III/II^, Ru^IV/III^, and Ru^V/IV^ at of 0.81, 1.03, and 1.42 V vs. normal hydrogen electrode (NHE), respectively. These potential values were very close to the corresponding redox potentials of 1/GC (Ru^III/II^ at 0.81 V, Ru^IV/III^ at 1.07 V, and Ru^V/IV^ at 1.42 V vs. NHE). Both CVs of 1/GC and poly-2@GC showed an onset potential for catalytic current at approximately 1.25 V vs. NHE, where the electrochemical Ru^V/IV^ oxidation started, implying that the [Ru^V^=O]^+^ species triggered O_2_ evolution in both cases [17]. Considering the backbone ligand of poly-2 tend to draw Ru(pda) units closer in a face-to-face manner of the active sides than the catalyst 1 in a dilute solution, the O-O bond formation via I2M is more likely to occur in the former situation than the latter one, leading to different catalytic water oxidation pathways by 1/GC and poly-2@GC.

To gain some more deep insights into the different O-O bond formation mechanisms between 1/GC and poly-2@GC, three key kinetic indicators were compared between catalytic water oxidation reactions by these two Ru(pda) catalytic systems [19]: (1) reaction orders of the catalyst, (2) reaction orders of the phosphate proton acceptor, and (3) deuterium kinetic isotope effects (*KIEs_H/D_*). In a WNA scenario as shown in Scheme 1, the catalytic reaction rate would show a first-order relationship with respect to the catalyst concentration, and increasing concentration of an effective proton acceptor, which promotes the formation of metal-hydroperoxide species (by atom-proton transfer), would facilitate the O-O bond formation. Meanwhile, the catalytic reaction rate usually displays a primary *KIE_H/D_* (>2) if the O-H bond cleavage is involved in the rate-determining step (RDS). In an I2M scenario, by contrast, the catalytic reaction rate would exhibit a second-order relationship depending on the catalyst concentration if the binuclear coupling is the RDS. At the same time, the overall rate of water oxidation should be insensitive to the proton acceptor concentration and deuterium substitution because proton transfer is not involved in the RDS.

Kinetic studies for water oxidation by Ru(pda) were firstly conducted by electrochemical methods in the homogeneous system (1/GC). The scan rate-dependent CVs of complex 1 ranging from 50 to 125 mV/s in aqueous sodium phosphate buffer solution (ionic strength = 1 M, Na_2_SO_4_) are shown in Figure S2a. The position of Ru^III/II^ redox peak at *E*_1/2_ = 0.55 V is independent of the scan rates; however, its peak current (*j*_p_) varies linearly to *υ*^1/2^ (Figure S2b). Meanwhile, as shown in Figure S2c and S2d, the peak current shows a linear relationship with the concentration of complex 1. This linear relationship is consistent with the Randles-Sevcik relation (Equation S1), indicating a diffusion-controlled redox process [20, 21].

**Figure 2:**
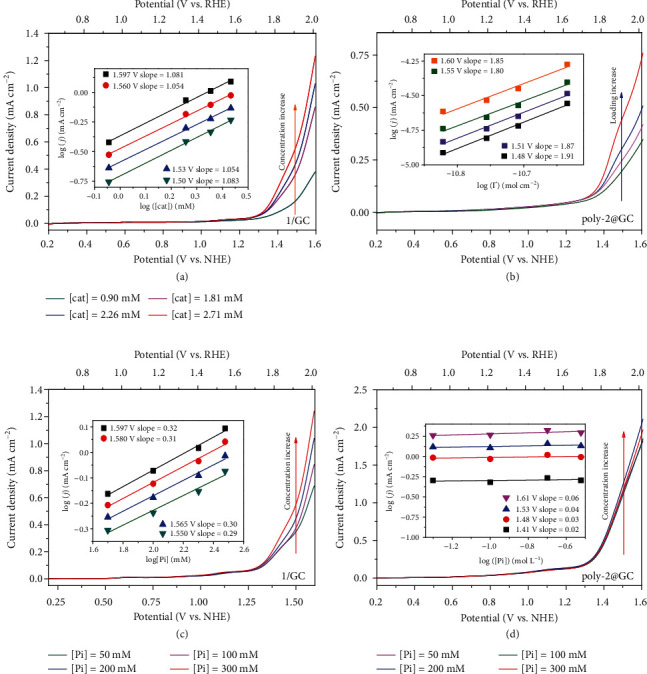
Linear sweep voltammograms (LSVs) of 1/GC (a) and poly-2@GC (b) at various concentrations of 1 or coverages (Γ) of 2, and LSVs of 1/GC (c) and poly-2@GC (d) at various concentrations of phosphate buffer. Inset plots show the logarithm relationship between the catalytic current density and the catalyst (a, b)/phosphate (c, d) concentration at selected potentials; the fitting slopes indicate reaction orders in catalyst concentration (*ρ*_[*cat*]_) (a, b) or phosphate concentration (*ρ*_[*pi*]_) (c, d).

The concentration-dependent catalytic currents were obtained from LSV curves of the 1/GC at various catalyst concentrations (as shown in Figure 2(a)). The apparent electrode reaction order of the catalyst (*ρ*_[*cat*]_^*j*_*cat*_^) based on the catalytic current can be calculated according to Equation S2, and all slopes approximately to 1 at different applied potentials indicate a single-site electrocatalytic pathway for the 1/GC system [22, 23]. The reaction rate constant of catalyst (*k*_*cat*_) can be obtained according to Equation S3.

The influence (*ρ*_[*cat*]_^*k*_*cat*_^) of the concentration of complex 1 on its *k*_*cat*_ for the water oxidation reaction can be evaluated (Equation S4). Because the reaction orders of the catalytic current (*ρ*_[*cat*]_^*j*_*cat*_^) and peak current (*ρ*_[*cat*]_^*j*_*p*_^) on the concentration of complex 1 are both 1; the calculated *ρ*_[*cat*]_^*k*_*cat*_^ is zero, suggesting the independence of the rate constant (*k*_*cat*_) on the concentration of the catalyst in a dilute solution. This is in agreement with the single-site WNA O-O bond formation mechanism [17]. In the case of a heterogeneous system catalyzed by the polymerized Ru(pda) (poly-2@GC) as described above, the high local catalyst concentration in the polymer (poly-2) backbone may shorten the intermolecular distance of Ru units and afford a Ru face-to-face environment, which is beneficial to the dinuclear I2M pathway. The Faraday efficiency of the complex 2 modified electrode (poly-2@GC) was measured (Equation S5) before the kinetic study [19]. As shown in Figure S3, the high Faraday efficiency of poly-2@GC indicated that the cumulative charges passing through the electrochemical system were almost quantitatively depleted in the water oxidation reaction, and therefore, the corresponding catalytic currents of the electrodes were directly applied for the kinetics analysis. Meanwhile, because this electrode reaction for the poly-2@GC system is not a catalyst diffusion-controlled process, the catalytic current density of poly-2@GC can represent the rate constant as well as the turnover frequencies of the catalyst on the surface. By adding the polarization times of electrochemical polymerization, poly-2@GC with various catalyst coverages (Γ) were obtained (Figure S4 and Equation S6). As shown in Figure 2(b), the catalytic current density (*j*_*cat*_) raised with an increasing Γ at the certain applied potential. According to Equation S2, S7, and S8, the reaction orders on the catalyst concentration (*ρ*_[*cat*]_^*j*_*cat*_^) were calculated to be 1.8~1.91 regarding applied potentials. Furthermore, the *ρ*_[*cat*]_^*k*_*cat*_^ is equal to the *ρ*_[*cat*]_^*j*_*cat*_^ as discussed above. The approximate value 2 of *ρ*_[*cat*]_^*k*_*cat*_^ indicates that the water oxidation reaction catalyzed by poly-2@GC is a second-order reaction on Ru(pda) unit. This observation is consistent with the I2M O-O bond formation pathway. Contrary to the homogeneous catalytic conditions, the *k*_*cat*_ of Ru(pda) on the electrode surface shows a strong correlation with the catalyst coverage. The difference of reaction kinetics between the homogenous and heterogeneous conditions confirms the promotion of I2M pathway by reducing intermolecular distance of Ru(pda) moieties on the electrode surface.

As described previously, atom-proton transfer (APT) can dramatically promote the rate of a WNA process [24]. As shown in Figure 2(c), the catalytic currents of 1/GC increase significantly with the increment of phosphate concentration; correspondingly, the electrode reaction order on phosphate (*ρ*_[*pi*]_^*j*_*cat*_^) can be defined and calculated to be 0.3 (Equation S9). The influence of phosphate concentration on the catalytic reaction rate constant *ρ*_[*pi*]_^*k*_*cat*_^ was evaluated in a similar manner (Equation S10). As shown in Figure S5a, the peak current (*j*_p_) does not change in the presence of various concentrations of phosphate, corresponding to a zero-order kinetic process, and *ρ*_[*pi*]_^*k*_*cat*_^ was then calculated as twice of *ρ*_[*pi*]_^*j*_*cat*_^ (approximately 0.6). Both *ρ*_[*pi*]_^*j*_*cat*_^ and *ρ*_[*pi*]_^*k*_*cat*_^ demonstrated the occurrence of phosphate-assisted atom proton transfer process during the electrocatalytic water oxidation reaction by complex 1 under homogeneous conditions. These findings are in line with the proposed WNA O-O bond formation mechanism. The [Pi]-dependent LSV curves of poly-2@GC were recorded by setting the catalyst coverage Γ and other parameters to constants and changing only the concentration of phosphate buffer (Na_2_SO_4_ salt is added to maintain the ionic strength to 1.0 M). As shown in Figure 2(d), the reaction rate of poly-2@GC did not rise by increasing the phosphate concentration, corresponding to a zero-order reaction on [Pi]. This suggests no involvement of the atom-proton transfer process in the RDS of water oxidation by poly-2@GC and a I2M pathway for Ru(pda) in the homopolymer form.

**Figure 3:**
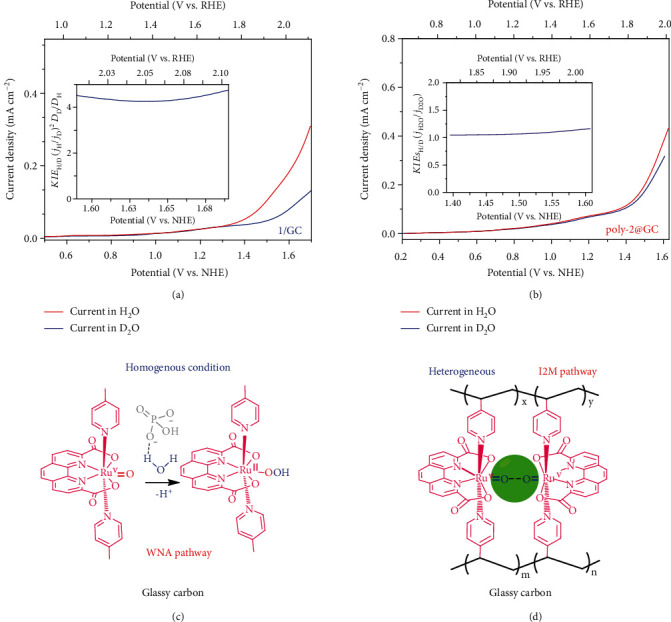
LSV curves of 1/GC (a) and poly-2@GC (b) in D_2_O and H_2_O (electrolyte: Na_2_SO_4_ anhydrous, 50 mM); the inset plots show the *KIE_H/D_* values as a function of the potential. Schematic diagram of the corresponding O-O bond formation mechanisms for (c) 1/GC and (d) poly-2@GC.

The H_2_O/D_2_O kinetic isotope effects (*KIEs*_H/D_) were further investigated to verify the proposed reaction pathways. As shown in Figure 3(a), *KIE*s_H/D_ based on the current of the electrode reaction (*KIEs*_*H*/*D*_^*j*_*cat*_^) was found to be ~2.3 according to *Equations*S11~S14 for 1/GC. The *KIEs*_H/D_ based on the catalyst rate constant (*KIEs*_*H*/*D*_^*k*_*cat*_^) can also be calculated (Equation S15) to be larger than 4 (Figure S6). The primary kinetic isotope effect on deuterium revealed that the O-H bond cleavage is involved in the O-O bond formation step for complex 1 in the 1/GC system, which is consistent with the WNA O–O bond formation mechanism. Under the heterogeneous catalytic conditions, LSV curves of poly-2@GC in H_2_O and D_2_O electrolytes were compared (Figure 3(b)), and no apparent H_2_O/D_2_O kinetic isotope effects (*KIEs*_*H*/*D*_ = 1.05 ~ 1.16) were observed in the potential range from 1.4 V to 1.6 V vs. NHE. These *KIEs_H/D_* values suggest that the O-H bond cleavage is not involved in the RDS of water oxidation catalysis by poly-2 and is consistent with the features of a radical coupling (I2M) mechanism.

Based on the experimental data, Figures 3(c) and 3(d) depicted the schemes for the O-O bond formation pathway of water oxidation by complex 1 in a homogeneous system and complex 2 on the electrode surface. As shown in Figure 3(c), the key points for the catalytic pathway of 1/GC are summarized as follows: (i) it is a single site electrochemical reaction in solution, (ii) water oxidation is assisted by the APT process using phosphate as the proton acceptor, and (iii) O-H bond cleavage is involved in the rate-determining O-O bond formation step. All these findings point to water oxidation mechanism via a single Ru site, and the WNA is the dominant O-O bond formation pathway for the 1/GC system. On the other hand, the O-O bond formation pathway of Ru(pda) in poly-2@GC system is proposed as Figure 3(d), which is a binuclear reaction with neither the assistance of phosphate nor the O-H bond cleavage in the rate-determining O-O bond formation step. The O-O radical coupling interaction of two metal-oxyl radicals is the dominant reaction pathway for Ru(pda) in poly-2@GC. The compact intermolecular distance of Ru centers on the electrode surface makes the approaching of two formal (pda)Ru^V^=O species overcome the steric hindrance of the pda ligand and benefits the I2M mechanism reaction rather than the WNA pathway of pristine Ru(pda)-type MWOC.

**Figure 4:**
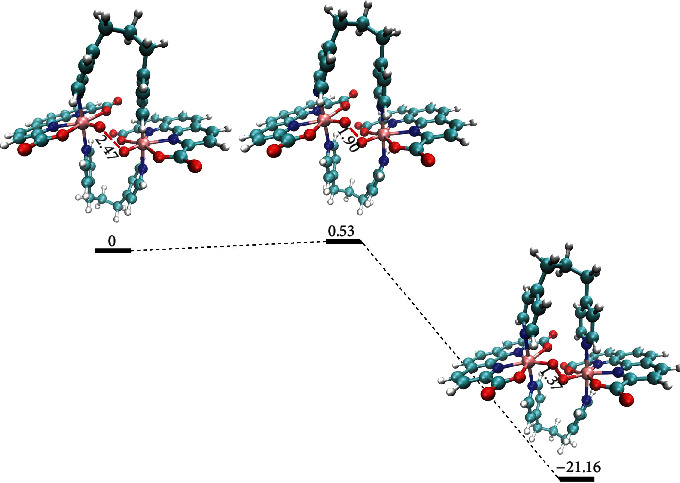
Calculated relative energy profile of O-O bond formation in the I2M pathway for a (pda) Ru^V^=O dimer complex. The relative energies are given in kcal mol^−1^ and bond distance in Å.

DFT calculations and molecular dynamics (MD) simulations were performed to further illustrate the reaction pathway of electrochemical driven water oxidation by Ru(pda) in poly-2@GC and properties of the catalyst. Our previous study has shown that the face-to-back configuration is also more favored than the face-to-face configuration of two (pda)Ru^V^=O, due to the hydrophobic pda backbone and the hydrophobic oxo, which results that Ru(pda) complex disfavored the binuclear I2M pathway for homogeneous water oxidation in aqueous solution [18]. The kinetic study on poly-2@GC showed a binuclear I2M reaction pathway. Hence, a supramolecular dimer with face-to-face configuration linked by two propyl groups was revisited as a reasonable initial structural model for the calculations. The calculated redox potentials of [Ru^II^ − OH_2_]/[Ru^III^ − OH_2_]^+^, [Ru^III^ − OH_2_]/[Ru^IV^ − OH]^+^, [Ru^IV^ − OH]/[Ru^V^ = O]^+^ are 0.63, 0.73, and 1.22 V at pH 7.0, respectively, which are close to the experimental values (Figure S7). For the key intermediate, [Ru^V^=O]^+^, MD simulations were performed to understand solvation properties of the oxo in the supramolecular dimer, by using the previously parameterized Ru(pda) model with new partial charge parameters [18]. The H-bond analysis and the radial distribution function (RDF) analysis (Figure S8) of oxygen atoms with water molecules showed that the oxo of the supramolecular dimer is also hydrophobic, the same as that in the molecular Ru(pda) catalysts. The high spin density of the oxo (0.69) from DFT calculations is similar to that of the oxo in molecular Ru(pda) (0.71). With the O-O bond formation step of two (pda)Ru^V^=O species, as shown in the relative free energy profile (Figure 4), the reaction is exergonic with a free energy of -21.16 kcal mol^−1^ from the prereactive to the product, accompanied by an activation free energy of 0.53 kcal mol^−1^ from the prereactive to the transition state. The low activation free energy of the (pda)Ru^V^=O coupling process, comparing to the activation free energy of 20.6 kcal mol^−1^ by WNA pathway [18], supports the experimental results that the Ru(pda) in poly-2@GC catalyzed water oxidation through the I2M mechanism. Once the catalysts overcoming the arrangement step and yielding the prereactive dimer state, the O-O bond is favored to form via coupling of two linked Ru(pda)^V^=O species. The theoretical evidence implied the possibility of switching the O-O bond formation pathway of the single-site Ru(pda) catalyst from the WNA mechanism to the I2M by manipulating the third coordination sphere, that is the hydrophobic/hydrophilic directionality of the catalysts. For instance, forcing the Ru(pda) catalyst to be a prereactive face-to-face dimer configuration via structural modification is a good strategy for I2M O-O bond formation pathway.

## 3. Discussion

Recently, several MWOCs based on first-row transition-metal such as Mn, Fe, Co, Ni, and Cu have been developed [25–35]. Although the catalytic mechanism and structure-activity correlation of these WOCs are still not known as much as that of Ru-based WOCs, they are very attractive for opening up the way to develop WOCs based on earth-abundant elements. The O-O bond formation is often the RDS for most WOCs; meanwhile, for most single-site molecular WOCs, the O_2_ is produced through WNA. How to improve the activity of single-site WNA WOCs attracts attention [11]. Among these, designing binuclear complexes from single-site catalysts has attracted much interest, owning to the potential possibility to obtain complexes that undergo the fast I2M pathway [7]. However, there is no clear experimental evidence that the reported binuclear complexes react via I2M mechanistic pathway. So far, most structure-activity studies of MWOCs have focused on the first and second coordination sphere surrounding the metal center of the catalysts (the chemical structure of the catalyst and proton transfer relay) [36–38]. A significant influence of intermolecular environments (the third coordination sphere) over the O-O bond formation pathway has rarely been reported. This is the first successful study on the switching of a WNA O-O bond formation pathway of a catalyst to a binuclear I2M pathway via manipulating the third coordination sphere of a catalyst. Our approaches might inspire related research on nonnoble metal WOCs-based water splitting devices in the future.

In summary, we studied the O-O bond formation mechanisms of electrochemical water oxidation by Ru(pda) type catalysts. Ru(pda) bearing 4-picoline axial ligands (1/GC) electrochemically catalyzed water oxidation through the water nucleophilic attack (WNA) pathway in a homogeneous system. When the intermolecular distance of the Ru(pda) type catalyst was forced to diminish and configurated in a face-to-face manner, by immobilizing complex 2, a Ru(pda) with 4-vinylpyridyl axial ligands, on glassy carbon electrode surface via electropolymerization (poly-2@GC), the O-O bond formation mechanism switched from WNA to intermolecular radical coupling (I2M). Kinetic studies, including the phosphate concentration and deuterium kinetic isotope effects upon reaction orders, revealed that Ru(pda) in the homocoupling polymer (poly-2) triggers two metal-oxyl radicals coupling interaction during water-oxidation catalysis. This I2M reaction pathway was also supported by DFT calculations. Our results suggest that the WNA O-O bond formation pathway of single-site MWOCs can switch to a binuclear I2M mechanism by manipulating the third coordination spheres of catalysts through structural modification. Our results provide a novel perspective for the design of more advanced water-oxidation catalysts by regulating the catalyst environment without changing the structures of catalysts.

## 4. Materials and Methods

### 4.1. Materials and Instrumentation

All commercial chemical reagents were used as received, and the water applied in this work was deionized using the Milli-Q technique. *cis*-Ru(DMSO)_4_Cl_2_ was prepared based on published works [39]. NMR spectra were measured by Bruker Advance 500 spectrometer. Mass spectra were collected on a Finnigan LCQ Advantage MAX mass spectrometer.

### 4.2. Synthesis

Complex 1 was prepared via a simple one-pot reaction. In a microwave vial (25 mL), 268 mg of pda =1,10-phenanthroline-2,9-dicarboxylate (pda), 1 mL of triethylamine, 484 mg of cis-Ru(DMSO)_4_Cl_2_, and 1 mL of 4-methyl pyridine were added together with MeOH (15 mL). The reaction mixture was heated at 100°C for 30 min in a microwave reactor (Biotage Initiator^+^). The solvent was then removed in the vacuum, and the residues were washed with diethyl ether. The crude product was purified by column chromatography over alumina using DCM : MeOH (100 : 3) as eluents, and complex 1 was obtained as a dark brown solid (567 mg, 38% yield) (38% yield). The 1H-NMR, MS, and elemental analyst were consistent with the work published previously [17]. Complex 2 was prepared via a simple one-pot reaction. In a microwave vial (25 mL), 268 mg of pda =1,10-phenanthroline-2,9-dicarboxylate (pda), 1 mL of triethylamine, 484 mg of *cis-*Ru(DMSO)_4_Cl_2_, and 1 mL of 4-vinyl pyridine were added together with MeOH (15 mL). The reaction mixture was heated at 100°C for 30 min in a microwave reactor (Biotage Initiator^+^). Methanol was then removed, and the residues were purified by chromatography using an Al_2_O_3_ column with DCM : MeOH (10 : 1) as an eluent, yielding a dark red powder as the desired product (468 mg, 30% yield) (30% yield). ^1^H-NMR (500 MHz, *d_4_*-CD_3_OD, Figure S1): 8.98 (d, *j* = 10, 1H), 8.91 (d, *j* = 10, 1H); 8.62 (s, 2H), 8.47 (d, *j* = 10, 2H), 8.27 (d, *j* = 10, 4H), 7.05 (dd, *j* = 10, 15, 2H), 6.43 (d, *j* = 20, 2H), 5.92 (d, *j* = 7.5, 2H); MS (ESI): calcd for 601.0633 (M+Na^+^), found *m*/*z*^+^ = 601.0633. Elemental analysis calculated to 52.57% C, 8.98% N, and 4.222% O.

### 4.3. Preparation of Poly-2@GC Electrodes

Before electropolymerization, all solutions were deaerated with Ar for 10 min. The preparation of poly-2@GC was based on the previous method [40]. Glassy carbon electrodes (diameter = 3 mm) were polarized from 0 to -2.4 V (vs. Ag/AgNO_3_) in successive cyclic scans in an acetonitrile solution containing 0.1 M TBAPF_6_ and 0.5 mM complex 2. The resulting poly-2@GC electrodes were rinsed with ethanol and dried under N_2_ flow.

### 4.4. Electrochemistry

Electrochemical measurements were carried out with a CH Instruments 660E electrochemical workstation at room temperature. A three-electrode configuration, where a glassy carbon was used as the working electrodes, a Ag/AgCl (3 M KCl) was used as the reference electrode, and a platinum wire was used as the counterelectrode, in a single compartment cell. All the measured potentials were converted to NHE according to a previous report [41]. All GC electrodes were polished with 1 *μ*m alumina powder before all experiments.

### 4.5. Computational Details

All DFT calculations for the estimation of Gibbs free energies were carried out with the Jaguar 8.3 program package by Schrödinger LLC [42]. Molecular geometries were optimized using Becke's three-parameter hybrid functional and the LYP correlation functional (B3LYP) with D3 correction of Grimme et al. with the LACVP∗∗ basis set [43–46]. To identify the transition states for O-O bond formation, we searched the potential energy surface by scanning the terminal O-O bond distance [Ru^V^=O•••O=Ru^V^] of the antiferromagnetic open-shell singlet. The thermochemical corrections for estimations of the Gibbs free energy barrier from the prereactive dimers were calculated at the B3LYP-D3/LACVP∗∗ level for both the prereactive dimers and transition state structures. Single-point energy corrections were performed with the B3LYP-D3 functional using the LACV3P∗∗++ basis set augmented with two *f*-functions on the metal. Based on the gas-phase optimized geometries, the implicit solvation energies were estimated by single-point calculations using the Poisson-Boltzmann reactive field implemented in Jaguar (PBF) in water. The Gibbs free energy was defined by the following equation G = E (B3LYP − D3/LACV3P∗∗++2f on Ru) + G_solv_ + ZPE + H_298_ − TS_298_ + 1.9 kcal mol^−1^ (the value 1.9 kcal mol^−1^ is a concentration correction to the free energy of solvation, which by default is calculated at 1 M (g) to 1 M (aq) in Jaguar).

MD simulations were performed with the Gromacs 5.1.4 MD software package [47]. A 20 ns MD run was performed in a 44 × 41 × 38 Å3 periodic box filled with TIP3P water molecules and two chloride ions to neutralize the charge [48]. In MD simulations, the resulting systems were subject to 100 000 steps of steepest descent minimization. The periodic boundary condition was applied in the simulation. The cutoff radius for the Lennard-Jones and electrostatic interactions were set to be 10 Å. For an accurate evaluation of the long-range Coulomb interactions, Particle Mesh Ewald (PME) summation method is used for electrostatic interactions beyond the cutoff [49]. The system was heated to 300 K in 100 ps by using a v-rescale thermostat for the canonical ensemble (NVT) simulations [50]. During this process, the Linear Constraint Solver (LINCS) algorithm was used to constrain all the bond lengths [51]. The isothermal isobaric ensemble (NPT) was used in the subsequent simulations, with the pressure set to 1 bar in 100 ps, controlled using a v-rescale thermostat and Parrinello-Rahman barostat [52]. Thereafter, the systems were simulated for 20 ns. Three repeated MD simulations with different initial velocities were also performed. A time step of 2.0 fs was used throughout the simulations.

## Supplementary Materials

Supplementary Materials Derivation of equations S1-S15. Figure S1: H-NMR spectrum of complex 2. Figure S2: scan rate-dependent CV measurements of complex 1. Figure S3: Ferriday efficiency of poly-2@GC. Figure S4: the voltammetric waves of poly-2@GC with various catalyst coverage. Figure S5: the concentration of phosphate-dependent CV measurements of 1/GC. Figure S6: diffusion constant measurements of complex 1 in D_2_O and the corresponding *KIEs_H/D_*. Figure S7: DFT optimized geometries of the intermediates. Figure S8: H-bond analysis, Figure S9: the RDF analysis. Table S1: the summary of the electrochemical data of several single-site Ru molecular catalysts. Data files S1-S7, the calculated geometries of intermediates and product and partial charge parameters.
