# Supplementary material for: Switching the O-O Bond Formation Pathways of Ru-pda Water Oxidation Catalyst by Third Coordination Sphere Engineering
Source: Research (Wash D C). 2021 Apr 13;2021:9851231. doi: 10.34133/2021/9851231 (PMC8061195; doi:10.34133/2021/9851231)
Supplement: Supplementary Materials — Derivation of equations S1-S15. Figure S1: H-NMR spectrum of complex 2. Figure S2: scan rate-dependent CV measurements of complex 1. Figure S3: Ferriday efficiency of poly-2@GC. Figure S4: the voltammetric waves of poly-2@GC with various catalyst coverage. Figure S5: the concentration of phosphate-dependent CV measurements of 1/GC. Figure S6: diffusion constant measurements of complex 1 in D2O and the corresponding KIEsH/D. Figure S7: DFT optimized geometries of the intermediates. Figure S8: H-bond analysis, Figure S9: the RDF analysis. Table S1: the summary of the electrochemical data of several single-site Ru molecular catalysts. Data files S1-S7, the calculated geometries of intermediates and product and partial charge parameters. [file 9851231.f1.docx]

Supporting Information

**Switching the O−O Bond Formation Pathways of Ru-pda Water Oxidation Catalyst by Third Coordination Sphere Engineering**

Yingzheng Li,^a†^ Shaoqi Zhan,^b.c†^ Lianpeng Tong,^d^ Wenlong Li,^a^ Yilong Zhao,^a^ Ziqi Zhao,^a^ Chang Liu,^a^ Mårten S. G. Ahlquist,^b^ Fusheng Li,*^a^ and Licheng Sun*^a,e,f^

a) State Key Laboratory of Fine Chemicals, Institute of Artificial Photosynthesis, DUT-KTH Joint Education and Research Centre on Molecular Devices, Dalian University of Technology, Dalian 116024, P. R. China. fusheng@dlut.edu.cn

b) Department of Theoretical Chemistry and Biology, School of Engineering Sciences in Chemistry Biotechnology and Health, KTH Royal Institute of Technology, 10691 Stockholm, Sweden

c) Department of Chemistry, University of California, Riverside, California 92521, United States

d) School of Chemistry and Chemical Engineering/Institute of Clean Energy and Materials, Guangzhou University, No. 230 Wai Huan Xi Road, Higher Education Mega Center, Guangzhou 510006, PR China

e) Department of Chemistry, School of Engineering Sciences in Chemistry, Biotechnology and Health, KTH Royal Institute of Technology, 10044 Stockholm, Sweden

f) Center of Artificial Photosynthesis for Solar Fuels, School of Science, Westlake University, 310024 Hangzhou, China. [sunlicheng@westlake.edu.cn](mailto:sunlicheng@westlake.edu.cn)

^†^These authors contributed equally to this work.

**EXPERIMENTAL SECTION**

Synthesis.

Scheme S1. Synthesis route of complex 1 and complex 2

**Electrochemical kinetics measurements of 1/GC**

For a diffusion-limited electron transfer reaction, the diffusion constant (*D*) can be calculated by ***eq. S1***, where *n* is the charge transfer number of the reaction (*n* = 1 for the Ru^III/II^ redox couple), *F* is the Faraday constant, *A* is the active area of the electrode, *v* is the scan rate, *R* is the gas constant, and *T* is the temperature.

$$j_{p}=0.446nFA[cat](nFvD/RT)^{1/2}\text{ }eq.\text{ S}1$$

When the concentrations of other species [*i*] keep constant, the reaction order of the catalyst on the catalytic current (*ρjcat [cat]*) can be calculated by ***eq. S2***, where *j*_cat_ is the catalytic current at a certain potential and [*cat*] is the concentration of the catalyst dissolved in the electrolyte.[[1-3](#_ENREF_1)]

*$\rho_{[cat]}^{j_{cat}}=\left\{\frac{\partial\log j_{cat}}{\partial\log[cat]}\right\}_{[i]\neq[cat]}\text{ } \text{ }eq. S2$*

Under homogeneous conditions, the rate constant (*k*_cat_) of the catalyst is given by ***eq. S3***,[[4](#_ENREF_4)] where *n’* is the charge transfer number of the catalytic reaction (*n’ =* 4 for water oxidation).

$$j_{cat}=n^{'}FA[cat](Dk_{cat})^{1/2}\text{ } \text{ }eq.\text{ S}3$$

Accordingly, the reaction order of the catalyst on the catalyst rate constant (*ρkcat [cat]*) can be evaluated according to ***eq. S4*** as combination ***eqs. S1-S3***, where $\frac{\partial\log j_{cat}}{\partial\log[cat]}$ is the reaction order of the catalyst related to the catalytic current; $\frac{\partial\log j_{p}}{\partial\log[cat]}$ is the reaction order of the catalyst related to the peak current.

*$\begin{matrix} \rho_{[cat]}^{k_{cat}}=\left\{\frac{\partial\log k_{cat}}{\partial\log[cat]}\right\}_{[i]\neq[cat]}\text{ } \\ \text{ = }\left\{\frac{2\partial\log\frac{j_{cat}}{j_{p}}}{\partial\log[cat]}\right\}_{[i]\neq[cat]}\text{ } \\ \text{ = }\left\{2\left(\frac{\partial\log j_{cat}}{\partial\log[cat]}-\frac{\partial\log j_{p}}{\partial\log[cat]}\right)\right\}_{[i]\neq[cat]}\text{ }eq. S4 \end{matrix}$*

**Faradaic Efficiency (FE) measurements of poly-2@GC**

Rotating ring disk (RRDE) LSV measurements were carried out on an RRDE-3A instrument (ALS Co., Ltd) and the CH instruments 760e bi-potentiostat (CH instrument, Inc. Austin) to calculate the Faradaic efficiency (*FE*) of **poly-2@GC** according to ***eq. S5***.[[5](#_ENREF_5)]

**

Where the number of electrons being transferred on the disk (*n_disk_*) corresponds to the four-electron process of the water oxidation reaction; the number of transferred electrons on the ring electrode (*n_ring_*) depends on the underlying mechanism for the ORR occurring at the ring; *N* is the collection efficiency for RRDE; *n_disk_*, *n_ring_*, and *N* are constants.[[6](#_ENREF_6)] RuO_2_ is a stable water oxidation catalyst that drives negligible side reactions; therefore, the value of *n_disk_*/(*N×n_ring_*) can be determined considering a 100% Faraday efficiency of RuO_2_ . The Faraday efficiency of the **poly-2@GC** electrode can thus be calculated.

**Electrochemical kinetics measurements of poly-2@GC**

The reaction order of the catalyst based on the catalytic current (*ρjcat [cat]*) can be calculated by ***eq. S2***, where the concentration of catalyst on the electrode surface, defined as the total amount of catalyst (*AΓ*) divided by the total volume (*V*) of the catalyst film, is equivalent to the catalyst coverage (*Γ*) divided by the thickness of the catalyst film (*h*). The *ρjcat [cat]* is given by ***eq. S6*** using the catalyst coverage as a variable.

If the electrode surface is not covered by more than one layer of catalysts, the thickness of the catalyst film (*h*) is a constant. The *ρjcat [cat]* can be then given by ***eq. S7***. Meyer and co-workers reported the *Γ* for Ru(bda) of monolayer was at 10^-10^ mol cm^-2^ level.^[^[^7^](#_ENREF_7)^]^ To ensure the catalyst coverage less than a monolayer, we controlled the catalyst coverage below 10^-10^ mol cm^-2^.

The catalyst coverage (*Γ*) could be calculated from the integrated current under voltammetric waves e.g. the coulombs by ***eq. S8,*** where Q is the current integration under the Ru^II/III^ of **poly-2@GC**, *F* is Faraday’s constant (96485 C), *n* is the number of electrons transferred (*n* = 1, in this case), and *A* is the area of the electrode.

*$\Gamma=\frac{Q}{nFA} eq. S 8$*

**Atom proton transfer measurements of 1/GC and poly-2@GC**

According to ***eq. S2***, the reaction order of the phosphate-assist atom proton transfer based on the catalytic current (*ρjcat [pi]*) for **1/GC** and **poly-2@GC** can be calculated by ***eq. S9***, where *[pi]* is the concentration of the phosphate buffer.

*$\rho_{[pi]}^{j_{cat}}=\left\{\frac{\partial\log j_{cat}}{\partial\log[pi]}\right\}_{[i]\neq[pi]}\text{ }eq. S9$*

The reaction order of *[pi]* based on the catalyst rate constant (*ρkcat [pi]*) can be obtained by ***eq. S10***, where $\frac{\partial\log j_{cat}}{\partial\log[pi]}$ is the reaction order of [pi] based on the catalytic current; $\frac{\partial\log j_{p}}{\partial\log[pi]}$ is the reaction order of *[pi]* based on the peak current.

*$\begin{matrix} \rho_{[pi]}^{k_{cat}}=\left\{\frac{\partial\log k_{cat}}{\partial\log[pi]}\right\}_{[i]\neq[pi]}\text{ } \\ \text{ = }\left\{\frac{2\partial\log\frac{j_{cat}}{j_{p}}}{\partial\log[pi]}\right\}_{[i]\neq[pi]} \\ \text{ = }\left\{2\left(\frac{\partial\log j_{cat}}{\partial\log[pi]}-\frac{\partial\log j_{p}}{\partial\log[pi]}\right)\right\}_{[i]\neq[pi]}\text{ } \text{ }eq. S10 \end{matrix}$*

**Kinetic isotope effects measurements of 1/GC and poly-2@GC**

Kinetic isotope effects (*KIEs*) were studied via electrochemical methods. *KIE*s_H/D_ based on the current of the electrode reaction (*KIEsjcat H/D*) was defined as ***eq. S11***, where *j*_H2O_ and *j*_D2O_ are the current density of **1/GC** and **poly-2@GC** in H_2_O or D_2_O solution at certain applied overpotential (*η*).

$$KIEs_{H/D}^{j_{cat}}=\left[\frac{j_{H_{2}O}}{j_{D_{2}O}}\right]_{\eta}\text{ } \text{ }eq.\text{ S11}$$

Because of the current density is the function of overpotential, the current densities *j*_H2O_ and *j*_D2O_ must be compared at the same overpotential in order to afford correct *KIEs_H/D_*.^[^[^8-9^](#_ENREF_8)^]^

The different free formation energy (Δ_formation_ *G*) of H_2_O (−237.18 kJ mol^−1^) and D_2_O (−243.49 kJ mol^−1^) lead to different water oxidation equilibrium potentials of 1.229 V_RHE_ and 1.262 V_RDE_ (reversible “deuterium” electrode, RDE).[[10-11](#_ENREF_10)]

The value of *η* can be corrected by ***eq. S12 and eq. S13*** for H_2_O and D_2_O solutions, respectively.

$$\eta^{H_{2}O}=E_{RHE}-1.229 V_{RHE}=E_{Ag/AgCl}^{read\text{ }in\text{ }H}+0.059pH+E_{Ag/AgCl}^{H}-1.229 V_{RHE}\text{ }eq.\text{ S12}$$

$$\eta^{D_{2}O}=E_{RDE}-1.262 V_{RDE}=E_{Ag/AgCl}^{read\text{ }in\text{ }D}+0.059pD+E_{Ag/AgCl}^{D}-1.262 V_{RDE}\text{ } \text{ }eq.\text{ S13}$$

Where $E_{Ag/AgCl}^{read in H}$ is the potential read by using the Ag/AgCl reference electrode in H_2_O solution; and $E_{Ag/AgCl}^{H}$ is the equilibrium potential of the Ag/AgCl couple in the NHE scales.[[3](#_ENREF_3), [12](#_ENREF_12)] $E_{Ag/AgCl}^{read in D}$ is the potential read by using the Ag/AgCl reference electrode in the D_2_O solution; and $E_{Ag/AgCl}^{D}$ is the equilibrium potential of the Ag/AgCl couple in the NDE scales (normal “deuterium” electrode).

The equilibrium potential of the deuterium couple (D_2_/D^+^) is different from that of the proton couple (H_2_/H^+^, the former is more negative than the latter by −0.013 V).[[10](#_ENREF_10)] Because 50 mM Na_2_SO_4_ (anhydrous) H_2_O solution and D_2_O solution were used as electrolytes, the pD could be calculated according to the following relationship: pD = pH^meter reading^+0.40 (pH^meter reading^ is the value reading by a pH meter,).[[13](#_ENREF_13)] Accordingly, the ***eq. S14*** was obtained:

$$\eta^{D_{2}O}=E_{RDE}-1.262 V_{RDE}=E_{Ag/AgCl}^{read\text{ }in\text{ }D}+0.059(pH+0.4)+E_{Ag/AgCl}^{D}-1.262 V_{RDE}\text{ }eq.\text{ S}14$$

In our experiments, the pH of 50 mM Na_2_SO_4_ (anhydrous) in H_2_O was measured as 6.87 using a pH meter, and the pH of 50 mM Na_2_SO_4_ (anhydrous) in D_2_O was measured as 6.92. To equalize the driving force in H_2_O solution and D_2_O solution for water oxidation, the difference between the potentials in H_2_O solution and in D_2_O solution ($E_{Ag/AgCl}^{read in D}$-$E_{Ag/AgCl}^{read in H}$), -19.5 mV, was corrected in the *KIEs_H/D_* calculation _._

For **1/GC**, *KIEs_H/D_* based on the rate constant (*k*_cat_) of catalyst ($KIEs_{_{H/D}}^{k_{cat}}$) can be evaluated according to ***eq. S11*** by combining ***eqs. S1*** and ***S3***, where *D*_D2O_ and *D*_H2O_ are the diffusion constants of the catalyst in D_2_O and H_2_O solutions, respectively. These diffusion constants can be measured according to ***eq. S1***.

$$KIEs_{_{H/D}}^{k_{cat}}=\left[\frac{k_{H_{2}O}}{k_{D_{2}O}}\right]_{\eta}=\left[\frac{j_{H_{2}O}}{j_{D_{2}O}}\right]^{2}_{\eta}\times\frac{D_{D_{2}O}}{D_{H_{2}O}}\text{ }eq.\text{ }S15$$

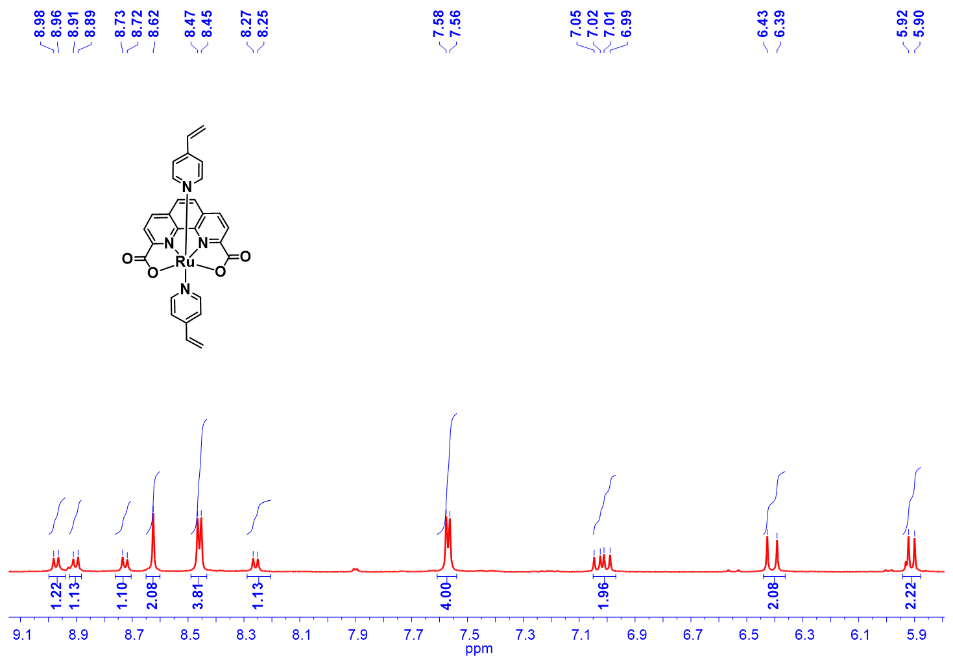


**Figure S1** H-NMR spectrum of complex **2.**


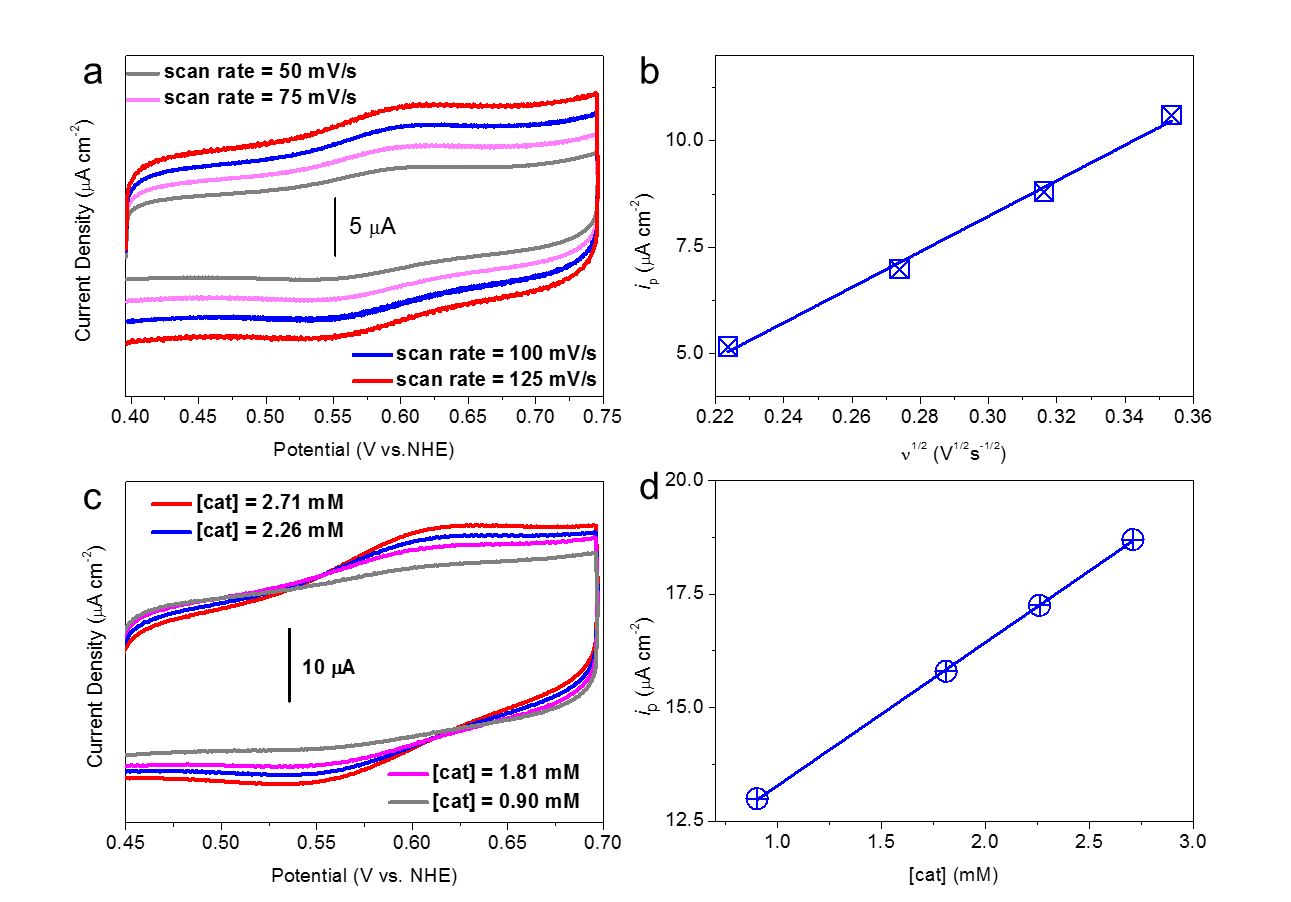


**Figure S2.** (a) Scan rates dependent CVs of complex **1** (2.52 mM) ranged from 50 to 125 mV s^-1^(100 mM sodium phosphate buffer, pH 7.0). (b) The corresponding plot of j_p_ as function of ν^1/2^. (c) the concentration dependent CVs of complex **1**, and (d) the corresponding plot of j_p_ as function of catalyst concentration.

**Figure S3**. Faraday efficiency plots as a function of potential, the plots inside show the rotating ring disk (RRDE) LSV curves of **poly-2@GC** and **RuO_2_@GC**.

For RRDE measurement, a Pt ring/GC disk electrode was used. Prior to use, the working electrode was polished mechanically with diamond down to an alumina slurry to obtain a mirror-like surface and then washed with deionized (DI) water, acetone and allowed to dry. Then, complex **2** was polymerized or copolymerized on the GC electrode as above mentioned method. All RRDE-LSV measurements were performed with 100 mM sodium phosphate buffer pH 7.0 as the supporting electrolyte (argon-purged) at 50 mV s^-1^ and 1500 rpm from 0.2 V to 1.5 V vs NHE. A potential of -0.25 V vs NHE was added on the Pt ring to detect the generation of O_2_. The Faraday efficiency can be derived from the ratio between disk and ring currents.In order to measure the value of n_disk_/(*N*×n_ring_), RuO_2_ was loaded on the GC disk. RuO_2_ powders were dispersed in the mixture solution of 1ml H_2_O, 0.25 mL 2-propanol and 10 ml 5% Nafion (ethanol solution) by sonication for 1 h, then, 2.5 μL of the above suspension were drop-casted to the pre-polished GC disk electrode, and dried at 50℃ for 15 min to evaporate the solvent.

**Figure S4**. The voltammetry waves (0.5 V vs NHE for 10 s and 0.9 V vs NHE for 40 s) of **poly-2@GC** with various catalyst coverage (*Γ*), the *Γ* was calculated according to ***eq. 14*** in the main text.


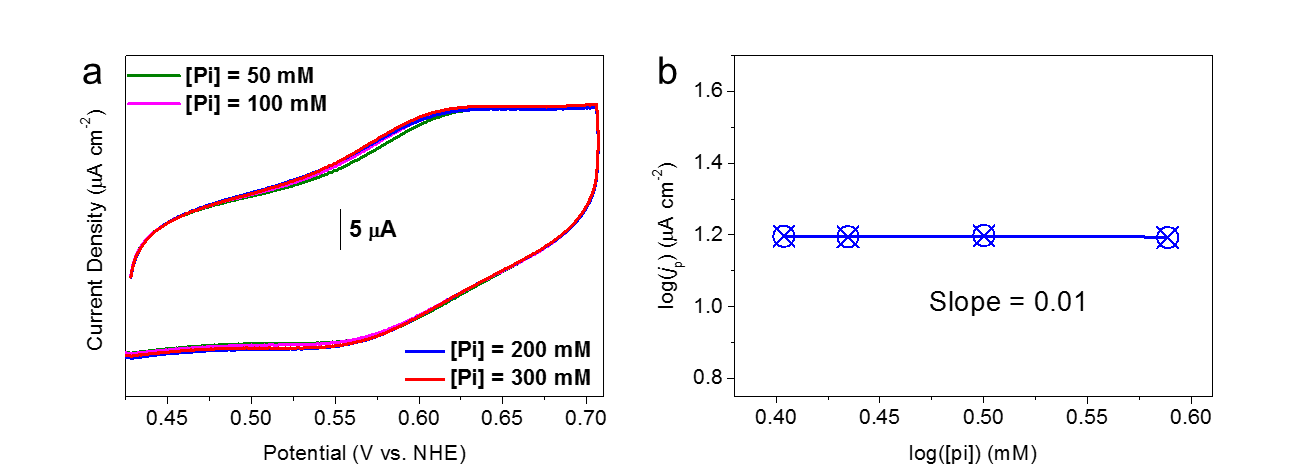


**Figure S5**. (a) CV curves of complex **1** at various concentrations of phosphate buffers range from 50 to 300 mM (the ion strength of phosphate buffer was fixed at 1 M by Na_2_SO_4_). (b) The corresponding plots of the logarithm of j_p_ as a function of the logarithm of phosphate concentration.


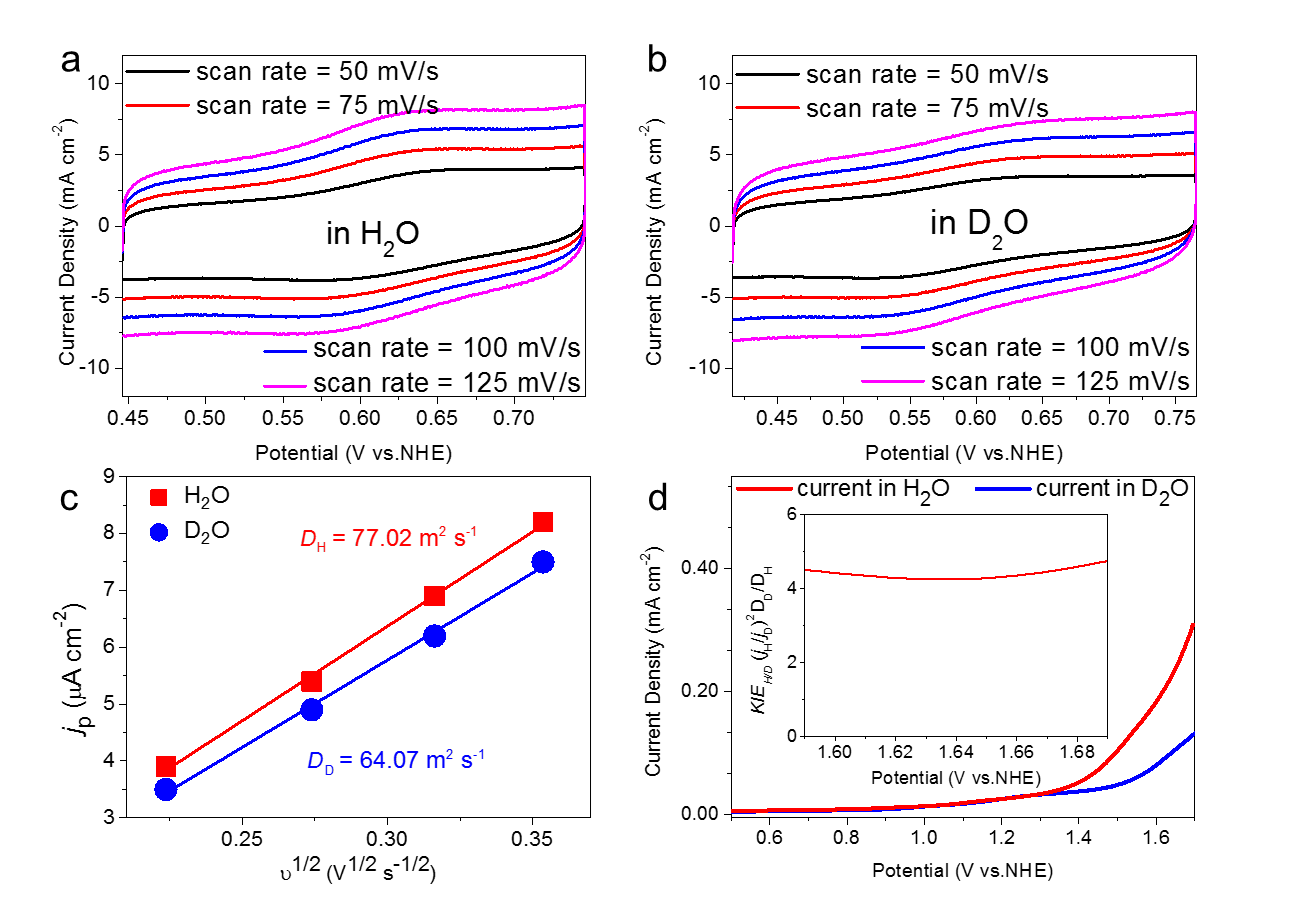


**Figure S6.** (a) Scan rates dependent CVs of **1/GC** ranged from 50 to 125 mV/s (50 mM Na_2_SO_4_ in H_2_O). (b) Scan rates dependent CVs of **1/GC** ranged from 50 to 125 mV/s (50 mM Na_2_SO_4_ in D_2_O). (c) The corresponding plot of j_p_ as a function of ν^1/2^ for calculation of diffusion constant. (d) LSV curves of **1/GC** in H_2_O and D_2_O (electrolyte: Na_2_SO_4_, 50mM), the plots inside show the KIEs values based on catalyst rate constant as a function of potential.


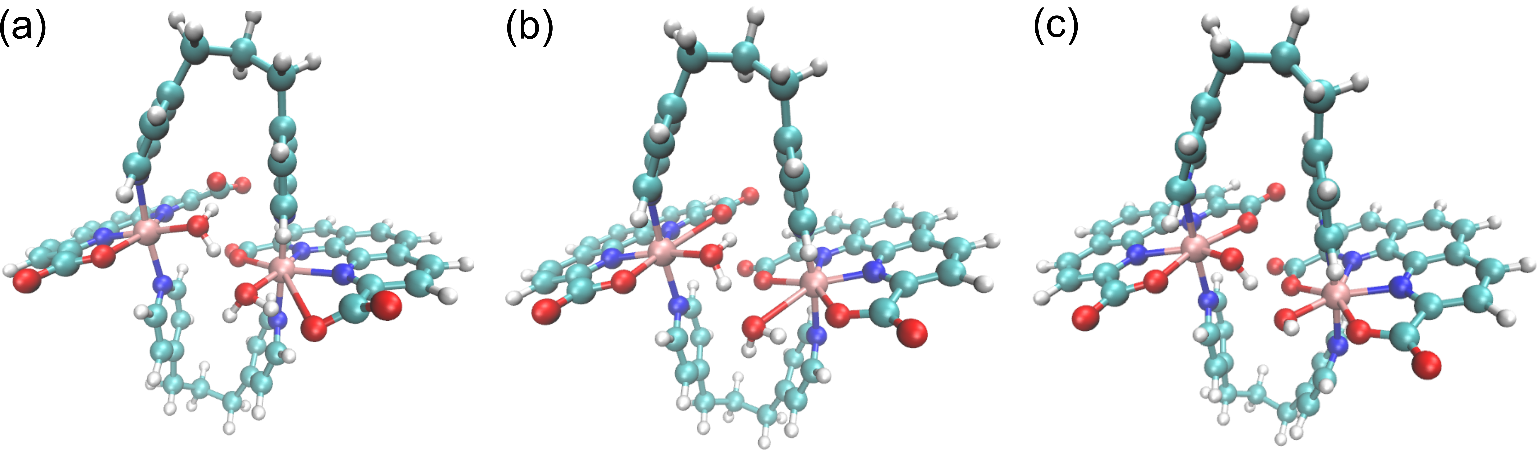


**Figure S7.** DFT optimized geometries of the [Ru^II^−OH_2_] (a), [Ru^III^−OH_2_]^+^ (b), and [Ru^IV^−OH]^+^ (c).


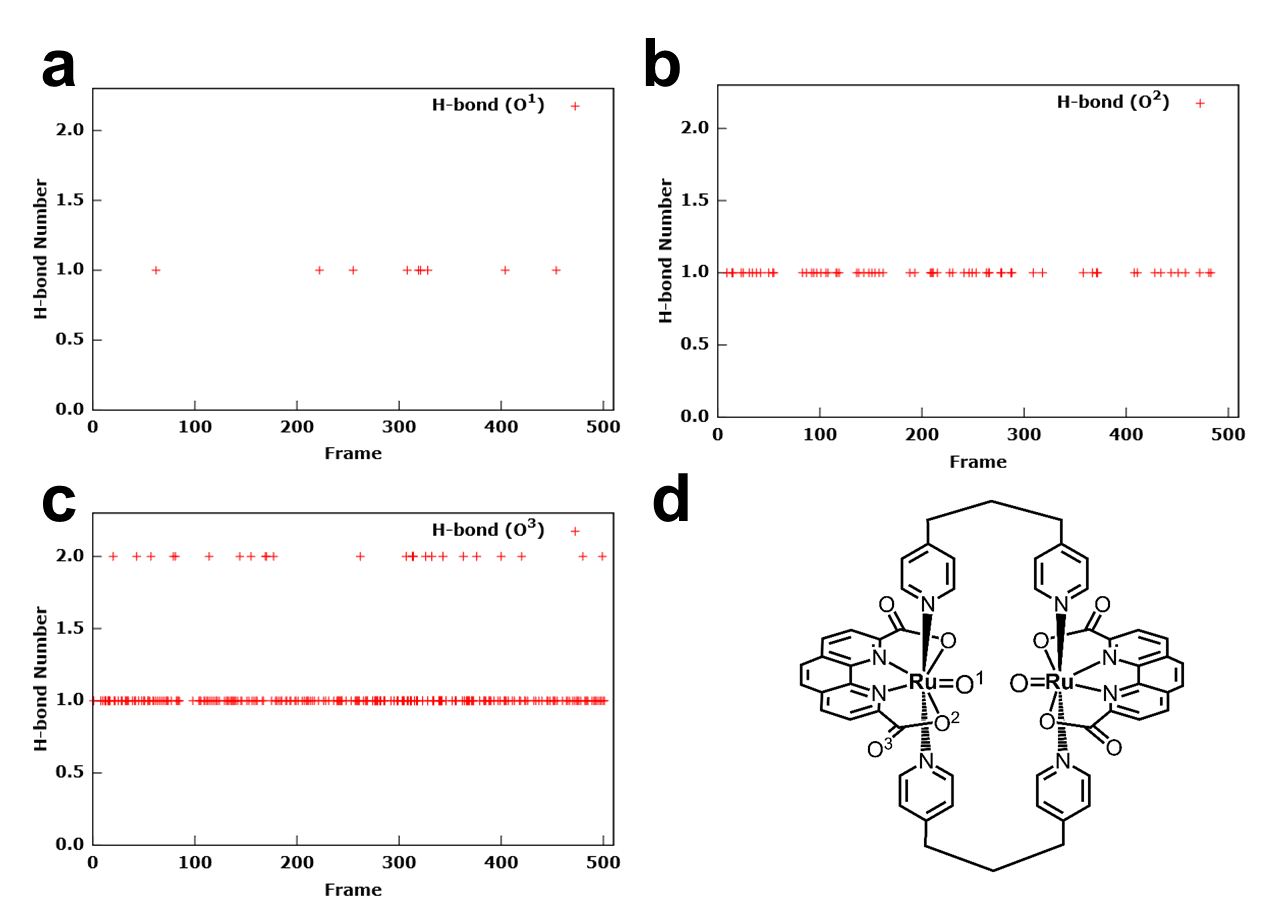


**Figure S8.** H-bond analysis between O^1^ (a), O^2^ (b), and O^3^ (c) of [Ru^V^=O]^+^ in the water phase and water. And the oxygen numbers are shown in the complexes of Ru^V^=O (d).


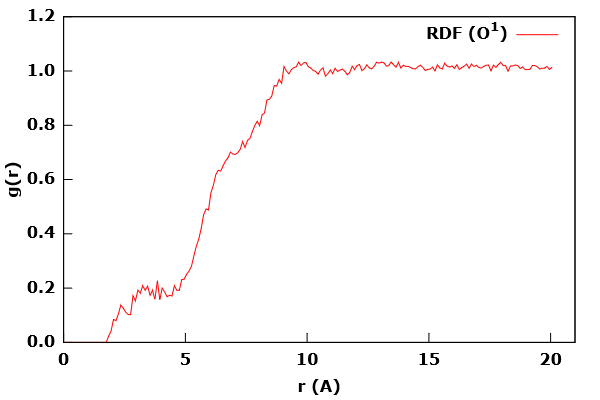


**Figure S9.** The Radial distribution function (RDF) analysis of O^1^ with water molecule.


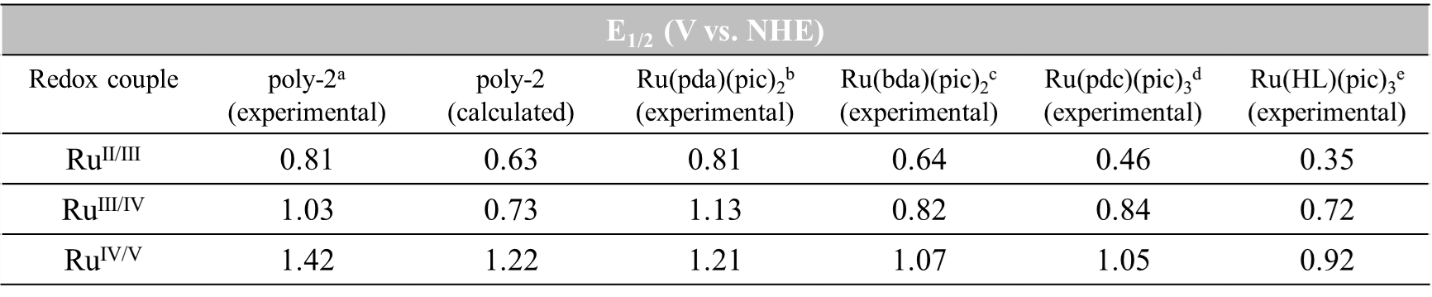


**Table S1.** Summary of the electrochemical data of several single-site Ru molecular catalysts.

^a^ Electrochemical measurement were performed in an aqueous sodium phosphate buffer solution (pH 7.0, ion strength = 1M) containing 10% (v/v) CH_3_CN. All potentials were obtained from DPV and are reported vs normal hydrogen electrode (NHE). Conditions: scan rate 0.1 V s^−1^, glassy carbon as a working electrode, a platinum foil as a counter electrode, and Ag/AgCl (3M KCl) as a reference electrode. Potentials were converted to NHE by using the [Ru(bpy)_3_]^3+^/[Ru(bpy)_3_]^2+^ couple as a standard (E_1/2_ = 1.26 V vs. NHE). ^b^ Electrochemical measurements were performed in an aqueous phosphate buffer solution (0.05 M, pH 7.0) containing 10% acetonitrile at a scan rate of 0.1 V s^−1^[[14](#_ENREF_14)]. ^c^ Electrochemical measurements were performed in an aqueous phosphate buffer solution (0.1 M, pH 7.0) at a scan rate of 0.1 V s^−1^ [[15](#_ENREF_15)]. ^d^ Electrochemical measurements were performed in an aqueous phosphate buffer solution (0.05 M, pH 7.0) containing 10% acetonitrile at a scan rate of 0.1 V s^−1^ [[16](#_ENREF_16)]. ^e^ Electrochemical measurements were performed in an aqueous phosphate buffer solution (0.1 M, pH 7.2) at a scan rate of 0.1 V s^−1^ [[17](#_ENREF_17)].

**Data file S1**

**Cartesian Coordinates in Å and Energies of the Calculated Geometries.**

**[Ru^II^-OH_2_]**

E (B3LYP-D3/LACV3P**++ 2f(Ru))(a.u.) = -3463.702

G_solv_ (kcal mol^-1^) = -41.664

ZPE (kcal mol^-1^) = 569.662

H_298_ (kcal mol^-1^) = 38.582

S_298_ (cal K^-1^ mol^-1^) = 321.73

Cartesian coordinates

Atom x y z

Ru1 -3.1469206537 -0.6184167199 5.7866021371

C2 -5.3960663962 -0.6900190827 2.2707821052

C3 -4.6739267141 -0.2936847788 3.4056988823

N4 -4.0357299197 -1.2066487517 4.1443926824

O5 -3.9064999790 1.2356994574 5.0660366016

C6 -4.5699694312 1.1293512738 3.9417999742

O7 -5.1127514765 2.0387952098 3.3233982310

C8 -7.5458878709 -1.1209817383 8.0171587593

C9 -7.0018854324 0.1188887172 7.6549176236

C10 -5.7441257046 0.1947482943 7.0689522111

N11 -4.9756827150 -0.8970819248 6.8502256515

C12 -5.4438508975 -2.0866177082 7.2871570026

C13 -6.7112819577 -2.2320573406 7.8461316862

C14 1.4628462097 0.6155185050 5.5530042141

C15 1.1110174355 -0.6735103583 5.1367097176

C16 -0.2197009544 -0.9851792080 4.9030475121

N17 -1.2109909326 -0.0828593831 5.0391226124

C18 -0.8694407811 1.1927034147 5.3234338528

C19 0.4413481912 1.5695199580 5.5864645313

H20 -5.8950289010 0.0831406268 1.6965760848

H21 -7.5771710022 1.0323310125 7.7771016888

H22 -5.3354649071 1.1338129714 6.7109799881

H23 -4.7783878045 -2.9354324662 7.2053903185

H24 -7.0292338566 -3.2280824121 8.1371020857

H25 1.8599672442 -1.4559709785 5.0571041852

H26 -0.5249782747 -1.9938204261 4.6558707869

H27 -1.6959700191 1.8900004578 5.3948744660

H28 0.6509379588 2.5984260366 5.8635155997

C29 -4.7984766154 -3.0016906352 2.7318194553

C30 -4.0844365760 -2.5277923388 3.8540178698

C31 -4.1260898867 -5.2575296855 3.3470229339

C32 -4.7988611994 -4.4170881087 2.5044335337

H33 -4.1322850385 -6.3287793242 3.1649445098

H34 -5.3424054482 -4.8123578336 1.6504281615

C35 -3.3715103471 -3.3877903496 4.7470316466

C36 -3.3913420533 -4.7758792623 4.4838761652

C37 -2.6723797004 -5.6073740811 5.3713414926

C38 -1.9701770478 -5.0277794716 6.4018395799

C39 -1.9596347345 -3.6242672511 6.5777668123

N40 -2.6798343183 -2.8086102586 5.7929456884

H41 -2.6760036250 -6.6849212930 5.2270164905

H42 -1.4011855209 -5.6031040715 7.1210736873

C43 -1.0074117078 -3.0596817205 7.6496965927

O44 -0.6957044029 -1.8421279726 7.5256146867

O45 -0.5923532097 -3.8730341446 8.5024760783

C46 -5.4571807423 -2.0344369702 1.9309111792

H47 -6.0140960059 -2.3581104450 1.0558443197

Ru48 -2.8858101533 -0.6972360549 10.8686580962

C49 -1.9124060453 -4.7115156784 11.4585016411

C50 -2.4062576607 -3.5071077722 10.9348139807

N51 -2.2669524738 -2.3775967621 11.6355386757

O52 -3.3142027678 -2.0650653078 9.2832854340

C53 -3.1613896781 -3.3117252759 9.6240557970

O54 -3.6201476074 -4.2885325868 9.0288127664

C55 -7.7063365889 -1.3687442449 11.3096848367

C56 -6.8426641949 -2.4679870872 11.3037541424

C57 -5.4748722990 -2.2706872613 11.3622904370

N58 -4.9143518912 -1.0460218363 11.4581151848

C59 -5.7443908116 0.0067280984 11.6400582514

C60 -7.1271765928 -0.1181330700 11.5462901657

C61 1.6229089996 0.5087038629 9.1481649506

C62 0.6314921144 1.4658335633 9.4087772305

C63 -0.5902974379 1.0801844823 9.9360604834

N64 -0.8987617594 -0.2118482935 10.1992876217

C65 0.0568521744 -1.1360544334 9.9740712845

C66 1.3149911828 -0.8095437792 9.4791714811

H67 -2.0239878964 -5.6068809788 10.8580617966

H68 -7.2229417170 -3.4778852860 11.1826722608

H69 -4.8086546192 -3.1168715336 11.2877002760

H70 -5.2609377935 0.9615714589 11.8306581117

H71 -7.7432188314 0.7727705916 11.6263216218

H72 0.8021419627 2.5163425072 9.1945648129

H73 -1.3626793106 1.8122758110 10.1340002239

H74 -0.2023608652 -2.1741604677 10.1294447427

H75 2.0163957330 -1.6172796249 9.2954958444

C76 -1.1679232483 -3.5074308168 13.4435722147

C77 -1.6888117443 -2.3388841413 12.8549860757

C78 -0.4948824389 -2.1432390669 15.3432952142

C79 -0.5586815480 -3.3665277290 14.7332093132

H80 -0.0233457385 -2.0486307919 16.3174946964

H81 -0.1423816587 -4.2449876110 15.2175930490

C82 -1.6443004883 -1.0516682095 13.4755832677

C83 -1.0262192201 -0.9519685995 14.7380572826

C84 -0.9543603508 0.3375888682 15.3145009787

C85 -1.4601020054 1.4081329054 14.6146470801

C86 -2.0861121501 1.2315716963 13.3540074523

N87 -2.1958779252 0.0194946601 12.7970304565

H88 -0.4857237846 0.4719918511 16.2859228425

H89 -1.3859007881 2.4248726399 14.9803323805

C90 -2.5704141314 2.5095035123 12.6316048195

O91 -3.5539526644 2.3944593699 11.8178441817

O92 -1.9630612157 3.5354087718 12.9460828185

C93 -1.2900337062 -4.7112288107 12.6986284141

H94 -0.8894650065 -5.6328041492 13.1119527479

C95 -9.1712886289 -1.5559235778 11.0040828114

H96 -9.6131180277 -2.2413940530 11.7392933134

H97 -9.7011420678 -0.6011782258 11.1031551791

C98 -9.4158281551 -2.1366141962 9.5908487549

C99 -9.0086986992 -1.2163636245 8.4215069037

C100 2.9767980512 0.8901504930 8.5842079084

H101 3.0420766785 1.9813604536 8.4940351798

H102 3.7304381770 0.6031982855 9.3297591468

C103 2.8868228020 0.9362777434 5.9470682352

C104 3.3921554956 0.2411084438 7.2385672691

H105 -10.4890184288 -2.3404470208 9.4975013743

H106 -8.9176082187 -3.1089447597 9.4990444776

H107 -9.5479989209 -1.5612603292 7.5279841608

H108 -9.3865772350 -0.2051184968 8.6180984490

H109 3.0941850963 -0.8141682378 7.2266438350

H110 4.4879429592 0.2520608222 7.2001805633

H111 3.0121017380 2.0214309373 6.0466105971

H112 3.5459166114 0.6233020253 5.1265115858

O113 -3.6430048326 1.0673290331 9.7630842686

H114 -3.6170333143 1.7407251821 10.5632717413

H115 -4.5657408590 0.9364320248 9.5027760504

O116 -2.1932295794 0.2027269588 7.5391907109

H117 -1.6192183102 -0.5836029144 7.7624320489

H118 -2.7463778533 0.4344872174 8.3032279163

**Data file S2**

**[Ru^III^-OH_2_]^+^**

E (B3LYP-D3/LACV3P**++ 2f(Ru))(a.u.) = -3463.215515

G_solv_ (kcal mol^-1^) = -126.233

ZPE (kcal mol^-1^) = 571.707

H_298_ (kcal mol^-1^) = 39.002

S_298_ (cal K^-1^ mol^-1^) = 325.199

Cartesian coordinates

Atom x y z

Ru1 -3.0962604673 -0.7734197292 5.8116997936

C2 -5.0418611630 -0.6701166787 2.0352732168

C3 -4.4217926934 -0.3548140046 3.2549872654

N4 -3.8783280810 -1.3085938122 4.0056324858

O5 -3.7814362523 0.9987032259 5.0846617401

C6 -4.3344402223 1.0202381901 3.8762344504

O7 -4.7623899600 2.0064469045 3.3175112065

C8 -7.5816722006 -1.2715063069 7.9484014364

C9 -7.0566711038 -0.0344079254 7.5417806981

C10 -5.8133742145 0.0392059470 6.9260502273

N11 -5.0462494423 -1.0533408966 6.7064364789

C12 -5.5265834732 -2.2453806187 7.1140612092

C13 -6.7663859714 -2.3877668871 7.7278547414

C14 1.5519820765 0.4544819136 5.5270837565

C15 1.1774131277 -0.8611745830 5.2174372339

C16 -0.1585805121 -1.1750758889 5.0416356240

N17 -1.1367814464 -0.2514022888 5.1521414268

C18 -0.7828494790 1.0371926864 5.3423999275

C19 0.5400616392 1.4185161305 5.5262607923

H20 -5.4626123823 0.1435766098 1.4542503728

H21 -7.6339810768 0.8780551232 7.6628732110

H22 -5.4081897033 0.9837442190 6.5830414371

H23 -4.8877651147 -3.1026325954 6.9706230623

H24 -7.0844451109 -3.3843331416 8.0161219757

H25 1.9218097163 -1.6487240235 5.1505231238

H26 -0.4748827498 -2.1919440118 4.8488295280

H27 -1.5966458664 1.7495934212 5.3975109462

H28 0.7676011838 2.4642482732 5.7072793696

C29 -4.5477242134 -3.0159786053 2.4326700782

C30 -3.9407130344 -2.6057724586 3.6355230369

C31 -3.9507506849 -5.3195046361 3.0032482169

C32 -4.5373069346 -4.4252498445 2.1473003067

H33 -3.9484667568 -6.3775192016 2.7600929427

H34 -4.9998639083 -4.7743555621 1.2290674778

C35 -3.3391658242 -3.5248830408 4.5332092546

C36 -3.3188682210 -4.8996818059 4.2266762229

C37 -2.6554162060 -5.7409247148 5.1537916888

C38 -2.0490241337 -5.1793171830 6.2631007874

C39 -2.1118499201 -3.7890914270 6.4773720886

N40 -2.7715922510 -2.9838992934 5.6438231924

H41 -2.6113113602 -6.8121012295 4.9780952643

H42 -1.5046783542 -5.7684718645 6.9921549226

C43 -1.3934814395 -3.0928188116 7.6179711232

O44 -1.4897712331 -1.8207332078 7.5408280433

O45 -0.7954764205 -3.7604780588 8.4724754891

C46 -5.1036236156 -1.9934887840 1.6224064002

H47 -5.5810367724 -2.2523051706 0.6818479776

Ru48 -2.9212570369 -0.3820423184 11.1854833676

C49 -2.0326688185 -4.5644694494 11.2645754318

C50 -2.5411141885 -3.3021219232 10.9083822529

N51 -2.3014091673 -2.2546422578 11.6855532769

O52 -3.5770491554 -1.6314870803 9.6027611165

C53 -3.3970918445 -2.9249874526 9.7131742791

O54 -3.8629635964 -3.7893191743 8.9832749447

C55 -7.6899284703 -1.1827199602 11.4576034825

C56 -6.7955042940 -2.2324741363 11.7051257353

C57 -5.4543147519 -1.9637696920 11.9142778073

N58 -4.9468726766 -0.7122990607 11.9004977557

C59 -5.8226261449 0.3130084720 11.8102888340

C60 -7.1792619110 0.1158227359 11.5810396894

C61 1.4529687065 0.6397262697 9.0124754817

C62 0.5127759398 1.6313380587 9.3326942312

C63 -0.6917612903 1.2980720978 9.9325881151

N64 -1.0257648785 0.0198024749 10.2342506171

C65 -0.1452843741 -0.9505174339 9.8962041383

C66 1.0789244614 -0.6776267056 9.3000602049

H67 -2.2251930473 -5.3960436631 10.5973307221

H68 -7.1321503865 -3.2644271812 11.6975784853

H69 -4.7535990197 -2.7735273390 12.0668270422

H70 -5.3967925733 1.3066159178 11.8892324637

H71 -7.8292044542 0.9798095909 11.4800628897

H72 0.7195122540 2.6770210790 9.1272199157

H73 -1.4186471726 2.0581983122 10.1878654191

H74 -0.4338165515 -1.9781552652 10.0601078520

H75 1.7239232624 -1.5164811417 9.0606415717

C76 -1.0379889245 -3.5784327424 13.2630046883

C77 -1.5830793575 -2.3650792071 12.8247337664

C78 -0.1322355377 -2.3520513081 15.1984235516

C79 -0.2941072200 -3.5238115476 14.4982647457

H80 0.4355441289 -2.3568394148 16.1234750907

H81 0.1478367645 -4.4393962761 14.8783694294

C82 -1.4172759269 -1.1589022452 13.5431983635

C83 -0.6938447893 -1.1033156496 14.7421026705

C84 -0.6022738734 0.1787614259 15.3492586282

C85 -1.2047136894 1.2800157752 14.7505239431

C86 -1.9070842629 1.1231769871 13.5397804498

N87 -1.9979145779 -0.0754178853 12.9814159080

H88 -0.0577564998 0.2974939402 16.2814864695

H89 -1.1511787422 2.2726506881 15.1849530591

C90 -2.6049401225 2.1966244722 12.7266572807

O91 -3.1546667115 1.7041650097 11.6197545576

O92 -2.6288971069 3.3549484600 13.0786108217

C93 -1.2844376518 -4.7037540420 12.4279893450

H94 -0.8811430011 -5.6746232174 12.7009145105

C95 -9.1205694208 -1.4685083430 11.0778271794

H96 -9.5682191800 -2.0883739141 11.8648622031

H97 -9.6958026089 -0.5366305513 11.0492907144

C98 -9.2825683919 -2.2186173318 9.7314551997

C99 -9.0059291757 -1.3823175561 8.4598389531

C100 2.8303950472 1.0075398173 8.4966652645

H101 2.8632434462 2.0844626244 8.2952000461

H102 3.5169071536 0.8449744561 9.3389858930

C103 2.9799850140 0.7768437729 5.8873771716

C104 3.4026412223 0.2426078311 7.2796502892

H105 -10.3218238809 -2.5583352098 9.6814868388

H106 -8.6697408456 -3.1279213103 9.7349885531

H107 -9.5817637329 -1.8317449861 7.6395794880

H108 -9.4211301105 -0.3761786555 8.5904046148

H109 3.1679991130 -0.8263685692 7.3510008457

H110 4.4935655866 0.3162365083 7.3344591294

H111 3.1463821792 1.8590545196 5.8460760304

H112 3.6436535221 0.3270162255 5.1393162077

O113 -4.2840231017 1.2439050361 9.3133569012

H114 -4.1015698776 1.9151944874 9.9960950426

H115 -5.2102983778 0.9775193177 9.3717849716

O116 -2.5214116267 0.4087918461 7.5244699156

H117 -2.0508467326 -0.3146515418 7.9978372768

H118 -3.2561416186 0.7084475563 8.1067001320

**Data file S3**

**[Ru^IV^-OH]^+^**

E (B3LYP-D3/LACV3P**++ 2f(Ru))(a.u.) = -3461.930239

G_solv_ (kcal mol^-1^) = -126.567

ZPE (kcal mol^-1^) = 558.249

H_298_ (kcal mol^-1^) = 37.743

S_298_ (cal K^-1^ mol^-1^) = 315.802

Cartesian coordinates

Atom x y z

Ru1 -2.9812516888 -0.9633341222 6.0313870508

C2 -5.0968681754 -0.4504723896 2.1681277611

C3 -4.4719160906 -0.2722699376 3.4154214413

N4 -3.9171373381 -1.2787661645 4.0733440942

O5 -3.6764520239 0.8898588874 5.2686230783

C6 -4.3415870212 1.0306197286 4.1449722232

O7 -4.8074408991 2.0674544432 3.7147917481

C8 -7.5032318909 -1.3986458234 8.0405381641

C9 -6.8959930999 -0.1636158813 7.7855588847

C10 -5.6280319604 -0.1021948113 7.2243020282

N11 -4.9290077076 -1.2117233567 6.9014380082

C12 -5.4875052530 -2.4103715095 7.1622676320

C13 -6.7509268009 -2.5381210639 7.7228113954

C14 1.6658152856 0.3068938946 5.5769780878

C15 1.3064325517 -1.0341505742 5.3771538111

C16 -0.0287364267 -1.3782001912 5.2668343858

N17 -1.0173184339 -0.4633841313 5.3372175698

C18 -0.6829143527 0.8402844571 5.4206254001

C19 0.6393469680 1.2528826772 5.5301537447

H20 -5.5260140977 0.4251543122 1.6926314442

H21 -7.4121187880 0.7654475978 8.0054878739

H22 -5.1540668056 0.8446624494 7.0016868183

H23 -4.8969973013 -3.2842424014 6.9317436664

H24 -7.1435073337 -3.5350387960 7.8965237079

H25 2.0619285935 -1.8136055773 5.3503583132

H26 -0.3267231583 -2.4121141038 5.1508852898

H27 -1.5075791929 1.5406385705 5.4576687919

H28 0.8543657695 2.3121820406 5.6296872865

C29 -4.5496148906 -2.8025271049 2.2865079445

C30 -3.9530325814 -2.5147182333 3.5278270948

C31 -3.8938735380 -5.1484851482 2.5863113306

C32 -4.4976012781 -4.1701376528 1.8402396290

H33 -3.8682172489 -6.1708505980 2.2215649982

H34 -4.9496323175 -4.4194418246 0.8849562494

C35 -3.3316491917 -3.5185612189 4.2956964825

C36 -3.2801203288 -4.8561403388 3.8544586979

C37 -2.6186870476 -5.7745147918 4.7065518935

C38 -2.0679193758 -5.3243918198 5.8963693404

C39 -2.1767267748 -3.9680692075 6.2445487271

N40 -2.7942277119 -3.0885597149 5.4596056834

H41 -2.5429924523 -6.8202048449 4.4225930063

H42 -1.5457968460 -5.9797344384 6.5846948288

C43 -1.6234843214 -3.3259338985 7.4805141040

O44 -1.9398676849 -2.0726738642 7.5496105947

O45 -0.9514584298 -3.9375196072 8.3092928797

C46 -5.1387032820 -1.7119050098 1.5990605263

H47 -5.6137789009 -1.8700157549 0.6353033943

Ru48 -3.0213788732 -0.2398929858 11.0593611176

C49 -1.9818892397 -4.4755387883 11.3278024786

C50 -2.5341753261 -3.2348222492 10.9688665259

N51 -2.2962637376 -2.1411269582 11.6789756361

O52 -3.7267623892 -1.6776176992 9.7182622068

C53 -3.4693757224 -2.9553304499 9.8325087762

O54 -3.9405888270 -3.8462367027 9.1440816537

C55 -7.7272339388 -1.0885575047 11.4991767260

C56 -6.8125264926 -2.0586696062 11.9371951714

C57 -5.4790163116 -1.7277093273 12.0838040032

N58 -5.0081925913 -0.4892526792 11.8229201333

C59 -5.8933498444 0.4812037364 11.5186808447

C60 -7.2484923126 0.2174348118 11.3647360880

C61 1.3963366062 0.6682052906 8.9219917416

C62 0.5098684939 1.6830393297 9.3116901117

C63 -0.7167075719 1.3732073608 9.8776339856

N64 -1.1206905358 0.0999088836 10.0815374533

C65 -0.3112424926 -0.8898024136 9.6475074359

C66 0.9331531913 -0.6438932396 9.0794140441

H67 -2.1967058332 -5.3228460771 10.6872639678

H68 -7.1314915429 -3.0796734204 12.1222512007

H69 -4.7516934214 -2.4729114829 12.3814820911

H70 -5.4836494012 1.4672493984 11.3486696062

H71 -7.9149705470 1.0284251590 11.0889563936

H72 0.7809282720 2.7281919741 9.1978284505

H73 -1.3922473002 2.1554045343 10.1984885335

H74 -0.6628852786 -1.9070963912 9.7326105377

H75 1.5277510277 -1.4973560050 8.7717944196

C76 -0.9491937801 -3.4137331672 13.2475263359

C77 -1.5382989336 -2.2223331604 12.7945469424

C78 -0.0594497117 -2.1401274757 15.1513121554

C79 -0.1863815853 -3.3222486013 14.4659467281

H80 0.5178860507 -2.1098474064 16.0698781223

H81 0.2913072855 -4.2193365668 14.8466921329

C82 -1.4101916114 -1.0123557801 13.4943615079

C83 -0.6789418484 -0.9237749679 14.6906397610

C84 -0.6380914603 0.3512497845 15.3096943322

C85 -1.2954561508 1.4181012658 14.7151159402

C86 -1.9900138333 1.2241144981 13.5084720925

N87 -2.0513236920 0.0343652257 12.9266206027

H88 -0.0936659483 0.4882266709 16.2393468890

H89 -1.2937938395 2.4165096724 15.1400794147

C90 -2.6914464398 2.2787933555 12.7110791244

O91 -3.1526457322 1.7852678706 11.5674879738

O92 -2.7921426516 3.4319022129 13.0609789303

C93 -1.1824752580 -4.5691993588 12.4569627681

H94 -0.7426186466 -5.5200826309 12.7430820334

C95 -9.1360766899 -1.4806210758 11.1387530627

H96 -9.5531338650 -2.0956178034 11.9451865968

H97 -9.7687046018 -0.5899799899 11.0582814202

C98 -9.2213770438 -2.2947199877 9.8210538188

C99 -8.9382852146 -1.4967395719 8.5229181940

C100 2.8090772296 1.0205670632 8.4917567279

H101 2.8519554697 2.0918987462 8.2621573790

H102 3.4295786052 0.8917941809 9.3900552990

C103 3.0881361502 0.6696900432 5.9148787362

C104 3.4818672865 0.2322164966 7.3480556633

H105 -10.2409297586 -2.6877333420 9.7560849555

H106 -8.5597965177 -3.1675474423 9.8833680408

H107 -9.5017314613 -1.9762499286 7.7122035568

H108 -9.3569751238 -0.4887870609 8.6187455519

H109 3.3097738097 -0.8445776640 7.4654806488

H110 4.5629486030 0.3749712846 7.4474897037

H111 3.2422155684 1.7489906976 5.8064504437

H112 3.7673425808 0.1776241381 5.2087915676

O113 -3.9184618996 0.7096838051 9.5295548156

H114 -3.6680792819 1.6423013496 9.6314902457

O116 -2.5409731947 0.2884058490 7.3522351038

H118 -3.1687259527 0.2769618452 8.1262447432

**Data file S4**

**I2M prereactive dimer**

E (B3LYP-D3/LACV3P**++ 2f(Ru))(a.u.) = -3460.608389

G_solv_ (kcal mol^-1^) = -127.983

ZPE (kcal mol^-1^) = 543.572

H_298_ (kcal mol^-1^) = 37.481

S_298_ (cal K^-1^ mol^-1^) = 311.428

Cartesian coordinates

Atom x y z

Ru1 -2.9243814308 -0.7224096806 6.1357468461

O2 -2.5921706195 0.4943854423 7.3250283382

C3 -5.1982620837 -0.5195076823 2.2916350622

C4 -4.4946864416 -0.2497534211 3.4803982435

N5 -3.9247926789 -1.2094007378 4.1916447981

O6 -3.6684392739 1.0622046947 5.2280727664

C7 -4.3206678630 1.1105630053 4.0885587242

O8 -4.7570583756 2.1139156856 3.5606334727

C9 -7.4999526505 -1.2562792959 7.9908919739

C10 -6.9245909093 -0.0141394601 7.6897987378

C11 -5.6321297470 0.0659445561 7.1913403653

N12 -4.8817717483 -1.0356161631 6.9749374756

C13 -5.3955772298 -2.2377157283 7.3026300134

C14 -6.6839536700 -2.3798866841 7.8015968088

C15 1.7050715866 0.4776302696 5.5649742987

C16 1.3256960471 -0.8593112712 5.3739346405

C17 -0.0133398556 -1.1853567421 5.2668873069

N18 -0.9832342823 -0.2498631415 5.3224392827

C19 -0.6304561759 1.0520016958 5.3751958911

C20 0.6968143772 1.4449778271 5.4899749062

H21 -5.6310372327 0.3214478581 1.7605177298

H22 -7.4856191815 0.9049384040 7.8262095839

H23 -5.1746451700 1.0158183228 6.9464372803

H24 -4.7544187208 -3.1004007799 7.2001787912

H25 -7.0373374300 -3.3797137486 8.0321267511

H26 2.0684470256 -1.6509644667 5.3599410256

H27 -0.3323540680 -2.2149207778 5.1718980309

H28 -1.4473337734 1.7648968376 5.3869694386

H29 0.9304101095 2.5014779478 5.5714513907

C30 -4.7242551912 -2.8719593005 2.6056572737

C31 -4.0337342271 -2.4898067549 3.7713402404

C32 -4.1456618919 -5.2033810283 3.0972800591

C33 -4.7594885740 -4.2765055172 2.2957740520

H34 -4.1883278167 -6.2568946296 2.8393009726

H35 -5.2873112211 -4.5973188466 1.4026776240

C36 -3.3986591433 -3.4423415700 4.5931520950

C37 -3.4333127720 -4.8159006689 4.2850693495

C38 -2.7555101633 -5.6797512934 5.1823716159

C39 -2.1020349420 -5.1461970572 6.2792944987

C40 -2.1279381088 -3.7572318102 6.4933962636

N41 -2.7637248751 -2.9299786556 5.6720227336

H42 -2.7515313795 -6.7507252664 5.0029460340

H43 -1.5688208061 -5.7550492102 7.0000723762

C44 -1.4486536683 -3.0374442238 7.6201793993

O45 -1.6890317096 -1.7680576634 7.6032799667

O46 -0.7483011805 -3.6314125952 8.4393306463

C47 -5.3173024386 -1.8267191566 1.8529003844

H48 -5.8605485492 -2.0567048175 0.9413537698

Ru49 -3.0421751754 -0.4090528303 10.8151507538

O50 -3.8070528071 0.3691461706 9.4681220311

C51 -2.0201165772 -4.7480350441 11.4064910896

C52 -2.5313873846 -3.5168023365 10.9622056116

N53 -2.3160168515 -2.3844049226 11.6223174381

O54 -3.6597706969 -2.0712588004 9.5428496580

C55 -3.3835000416 -3.3175886830 9.7448390925

O56 -3.7576968510 -4.2628234048 9.0517165624

C57 -7.7775309539 -1.1494425406 11.3561872886

C58 -6.8904578084 -2.1764751486 11.7104605119

C59 -5.5380981636 -1.9095039793 11.8173376182

N60 -5.0320642414 -0.6770387589 11.6058750061

C61 -5.8842269847 0.3470088480 11.3915873823

C62 -7.2524882048 0.1440214369 11.2717609724

C63 1.4003407147 0.7217268086 8.9199145220

C64 0.3518345457 1.6444294202 9.0373584188

C65 -0.8747067974 1.2608832250 9.5606594957

N66 -1.1072150346 -0.0022408415 9.9774350087

C67 -0.1309372399 -0.9192171013 9.8287262665

C68 1.1169762021 -0.5929936991 9.3138517333

H69 -2.2358260884 -5.6260512365 10.8087025252

H70 -7.2446987035 -3.1927748862 11.8526308096

H71 -4.8310587963 -2.6969755341 12.0409149807

H72 -5.4309751584 1.3225179880 11.2550546667

H73 -7.8943375137 0.9921738911 11.0561187555

H74 0.4860811361 2.6781067216 8.7349463289

H75 -1.6936279123 1.9608469585 9.6650880491

H76 -0.3618579462 -1.9410570612 10.0899594019

H77 1.8605632258 -1.3793159756 9.2328188154

C78 -1.0311411844 -3.6002360959 13.2929942285

C79 -1.5884286717 -2.4217982588 12.7617905496

C80 -0.1300893766 -2.2701185205 15.1437794666

C81 -0.2862998926 -3.4786615646 14.5171581964

H82 0.4348396007 -2.2087367259 16.0685282153

H83 0.1547976922 -4.3729300447 14.9459341825

C84 -1.4278290734 -1.1786847858 13.4072238847

C85 -0.7021669734 -1.0640707053 14.6077304304

C86 -0.6104643020 0.2352691440 15.1687218355

C87 -1.2189099622 1.2988565319 14.5249796416

C88 -1.9157909580 1.0737932728 13.3231717949

N89 -2.0187215788 -0.1325867614 12.7884447894

H90 -0.0650982032 0.3877670985 16.0955959045

H91 -1.1831990242 2.3148750852 14.9031780999

C92 -2.5976800895 2.1351240183 12.5099940784

O93 -3.1379984125 1.6442857729 11.4175849093

O94 -2.6176560098 3.3004453295 12.8556048101

C95 -1.2661184784 -4.7945491187 12.5660905859

H96 -0.8578522052 -5.7353839057 12.9241019393

C97 -9.2049374034 -1.4684837769 10.9994719503

H98 -9.6391423625 -2.1106316850 11.7748723256

H99 -9.8029011637 -0.5517844832 10.9708574590

C100 -9.3218561501 -2.2055868006 9.6406326680

C104 -8.9591907478 -1.3663343696 8.3924780134

C107 2.7879199335 1.1615062678 8.4912211661

H108 2.7642539179 2.2265038229 8.2335532288

H109 3.4187683138 1.0874883698 9.3865422372

C110 3.1288600575 0.8141408572 5.9182836621

C114 3.4942452000 0.3770890726 7.3597525972

H107 -10.3636222436 -2.5248683395 9.5354067035

H110 -8.7237326955 -3.1240876581 9.6696272785

H111 -9.4755517144 -1.8147214718 7.5334938481

H112 -9.3805162833 -0.3599462993 8.4968441350

H113 3.3154073827 -0.6988628597 7.4726798942

H114 4.5730355815 0.5202946237 7.4774124609

H115 3.3052366115 1.8886686235 5.8050483529

H116 3.8057205562 0.3029102172 5.2235065351

**Data file S5**

**I2M transition state**

E (B3LYP-D3/LACV3P**++ 2f(Ru))(a.u.) = -3460.601843

G_solv_ (kcal mol^-1^) = -129.701

ZPE (kcal mol^-1^) = 543.524

H_298_ (kcal mol^-1^) = 36.945

S_298_ (cal K^-1^ mol^-1^) = 307.145

Cartesian coordinates

Atom x y z

Ru1 -2.9983599331 -0.8304997131 6.2861268360

O2 -2.5770878101 0.2304252473 7.6643136674

C3 -5.1392772064 0.0271203899 2.4783518893

C4 -4.4697108364 0.0981158980 3.7135886575

N5 -3.9660535830 -0.9764934418 4.2998357371

O6 -3.6955721787 1.0915081608 5.6765690216

C7 -4.2631139247 1.3473965807 4.5183324665

O8 -4.6120340328 2.4403876840 4.1204185140

C9 -7.5647571580 -1.7345234127 8.0269708025

C10 -7.1278972449 -0.4642944291 7.6286295934

C11 -5.8327986634 -0.2732336548 7.1726849875

N12 -4.9495461367 -1.2904672895 7.0737628914

C13 -5.3339192812 -2.5136699928 7.4901195530

C14 -6.6158831644 -2.7639700004 7.9660050345

C15 1.6390797050 0.4475489293 5.6508049906

C16 1.2313786200 -0.8535806462 5.3214953896

C17 -0.1155359353 -1.1628026188 5.2784176998

N18 -1.0699592482 -0.2439583755 5.5309702327

C19 -0.7000399714 1.0384618675 5.7276406599

C20 0.6366237737 1.4128726410 5.7795957127

H21 -5.5175106015 0.9524992897 2.0570058126

H22 -7.7963703486 0.3900201595 7.6678464630

H23 -5.4708765679 0.7063793286 6.8894995525

H24 -4.5952255589 -3.3015846976 7.4782852009

H25 -6.8603242121 -3.7732310254 8.2815832554

H26 1.9617656746 -1.6376162881 5.1484697831

H27 -0.4497271893 -2.1718121780 5.0764057573

H28 -1.5047194311 1.7440524128 5.9007841940

H29 0.8847540797 2.4496860914 5.9826770305

C30 -4.7680208178 -2.3646063132 2.4733855104

C31 -4.1034654184 -2.1790454782 3.6992379301

C32 -4.2634687827 -4.7607723367 2.6339689748

C33 -4.8344623455 -3.7097609920 1.9645954379

H34 -4.3266561591 -5.7637087975 2.2230257675

H35 -5.3479715782 -3.8819903980 1.0234773769

C36 -3.5087869860 -3.2578148242 4.3835481859

C37 -3.5630478705 -4.5702668393 3.8765996703

C38 -2.9067796749 -5.5689458555 4.6391545281

C39 -2.2436545325 -5.2135790303 5.8028921105

C40 -2.2438993617 -3.8711811680 6.2170939630

N41 -2.8693591939 -2.9243750012 5.5256935318

H42 -2.9191818918 -6.6018721148 4.3032848039

H43 -1.7151491248 -5.9306450553 6.4210618039

C44 -1.5379953207 -3.3166901194 7.4196008838

O45 -1.7753418858 -2.0510107831 7.5679304188

O46 -0.8111562341 -3.9946204850 8.1366412817

C47 -5.2968748808 -1.2010133154 1.8590109302

H48 -5.8179703836 -1.2781534557 0.9096748532

Ru49 -2.9451970673 -0.4638804823 10.6876354849

O50 -3.7165338268 0.1163690821 9.1804477533

C51 -1.8628481131 -4.6480478920 11.8375591210

C52 -2.3946284036 -3.4998684578 11.2278625357

N53 -2.2343215533 -2.2896613707 11.7528386911

O54 -3.5193533497 -2.2510011445 9.6363391580

C55 -3.2228751381 -3.4657747173 9.9770047647

O56 -3.5822780520 -4.4798765671 9.3895453472

C57 -7.7137499373 -1.1341866453 11.2814769104

C58 -6.8153480408 -2.0865310432 11.7846877927

C59 -5.4633394330 -1.8013584439 11.8280217430

N60 -4.9663672768 -0.6187923300 11.4112731948

C61 -5.8266223889 0.3518523574 11.0413655314

C62 -7.1961187434 0.1284838922 10.9793131991

C63 1.6480099715 0.3148968891 8.9578804366

C64 0.7245158315 1.3438409151 9.1859499508

C65 -0.5506480111 1.0594733734 9.6493056827

N66 -0.9514875622 -0.2024428054 9.9180395225

C67 -0.0942087685 -1.2125062221 9.6671238525

C68 1.1931397860 -0.9896491138 9.1918039201

H69 -2.0307747785 -5.6009113371 11.3481233903

H70 -7.1614075531 -3.0671938520 12.0958675054

H71 -4.7494578163 -2.5403690510 12.1662713956

H72 -5.3807767438 1.2930021011 10.7400845898

H73 -7.8443353517 0.9279668181 10.6350117055

H74 0.9931026180 2.3802079743 9.0067692499

H75 -1.2848803690 1.8394086519 9.8025853082

H76 -0.4515571609 -2.2211181832 9.8145397755

H77 1.8328295726 -1.8483605624 9.0145606138

C78 -0.9915042287 -3.2461496240 13.6093397952

C79 -1.5560676334 -2.1603898285 12.9136589997

C80 -0.2533298879 -1.6705473888 15.3368309722

C81 -0.3212792464 -2.9486854844 14.8471731987

H82 0.2497498514 -1.4784182381 16.2791871388

H83 0.1291229709 -3.7656197518 15.4017477896

C84 -1.4775038712 -0.8449041347 13.4129513063

C85 -0.8424657201 -0.5605257923 14.6353398159

C86 -0.8582310180 0.7933825027 15.0566199125

C87 -1.4809535457 1.7439239361 14.2657346702

C88 -2.0740502017 1.3531472965 13.0511995710

N89 -2.0712407844 0.0937715764 12.6435448702

H90 -0.3875091883 1.0754510435 15.9938707130

H91 -1.5313763577 2.7934983907 14.5353580344

C92 -2.7475728229 2.2745078690 12.0772877379

O93 -3.1162575993 1.6418760769 10.9848898127

O94 -2.8964650332 3.4586129130 12.3015387543

C95 -1.1558033475 -4.5260231596 13.0228973361

H96 -0.7364188243 -5.4020140237 13.5089759000

C97 -9.1460423844 -1.5156023123 11.0088678304

H98 -9.5749027620 -1.9698399508 11.9101125838

H99 -9.7416694637 -0.6237016993 10.7877888193

C100 -9.2788529914 -2.5341361095 9.8473842266

C104 -9.0063922929 -1.9786705373 8.4294928469

C107 3.0778472907 0.6194130801 8.5539554876

H108 3.2235065537 1.7057336443 8.5476470687

H109 3.7233405847 0.2326189231 9.3531130686

C110 3.0914019443 0.7359312213 5.9324955791

C114 3.5970954931 0.0339890441 7.2194748421

H107 -10.3069172659 -2.9103898474 9.8573541214

H110 -8.6337623338 -3.3996009960 10.0410068926

H111 -9.4192367611 -2.6991026972 7.7115477703

H112 -9.5780829292 -1.0537447367 8.2916040672

H113 3.3736397450 -1.0386700519 7.1676405598

H114 4.6886104831 0.1180742171 7.2317234323

H115 3.2576210286 1.8158686686 6.0105556601

H116 3.6978949152 0.3807552571 5.0907771981

**Data file S6**

**I2M product state**

E (B3LYP-D3/LACV3P**++ 2f(Ru))(a.u.) = -3460.635262

G_solv_ (kcal mol^-1^) = -129.787

ZPE (kcal mol^-1^) = 544.106

H_298_ (kcal mol^-1^) = 36.635

S_298_ (cal K^-1^ mol^-1^) = 304.114

Cartesian coordinates

Atom x y z

Ru1 -3.0927819477 -0.9149555297 6.3287084593

O2 -2.6654564230 0.0961432971 7.9266065450

C3 -5.1470435099 0.4038929379 2.6387727853

C4 -4.5107576007 0.3336279424 3.8914679350

N5 -4.0355253268 -0.8067873147 4.3657969807

O6 -3.7936460386 1.0641201194 5.9849944622

C7 -4.3054007202 1.4766764940 4.8446034778

O8 -4.6155232216 2.6176683764 4.5698529341

C9 -7.5891796587 -2.1798815523 8.0508216506

C10 -7.3070590705 -0.9200368723 7.5087372091

C11 -6.0318742871 -0.6173976566 7.0579768062

N12 -5.0231442047 -1.5137274403 7.0894689957

C13 -5.2681878724 -2.7276435215 7.6225830491

C14 -6.5203793576 -3.0842236412 8.1111875210

C15 1.5422281188 0.4347252852 5.7784950162

C16 1.1093785700 -0.8047962226 5.2839674298

C17 -0.2404978998 -1.1000951054 5.2519783274

N18 -1.1792502906 -0.2268734886 5.6735464640

C19 -0.7898420754 1.0114035384 6.0390462010

C20 0.5519699198 1.3704050621 6.0893257808

H21 -5.5040837223 1.3739957934 2.3093069238

H22 -8.0792273994 -0.1597439565 7.4416877521

H23 -5.7878761530 0.3682039846 6.6873535335

H24 -4.4397600964 -3.4171177914 7.6969233448

H25 -6.6457178265 -4.0755989987 8.5356514141

H26 1.8241590578 -1.5574502527 4.9662007443

H27 -0.5890160566 -2.0687269361 4.9176244467

H28 -1.5787847428 1.6875111494 6.3470265644

H29 0.8154936757 2.3662576772 6.4306160442

C30 -4.7976624856 -1.9783480238 2.3774525005

C31 -4.1660166787 -1.9336683377 3.6329382444

C32 -4.2931345044 -4.3799715048 2.2890987394

C33 -4.8505773592 -3.2611142486 1.7251748357

H34 -4.3435833185 -5.3311368710 1.7679380285

H35 -5.3398982994 -3.3301451115 0.7583518776

C36 -3.5863128115 -3.0799148843 4.2085055517

C37 -3.6197690721 -4.3288515033 3.5608060062

C38 -2.9640743676 -5.3986829865 4.2204093683

C39 -2.3176524269 -5.1649877386 5.4247860392

C40 -2.3363720405 -3.8764692231 5.9836899247

N41 -2.9662160269 -2.8677029369 5.3901423315

H42 -2.9585671330 -6.3889953711 3.7743047126

H43 -1.7830420712 -5.9399641985 5.9635012312

C44 -1.6398610550 -3.4337894511 7.2371913451

O45 -1.8990686250 -2.1860148481 7.5056228932

O46 -0.8988220642 -4.1576517293 7.8859157808

C47 -5.2997614195 -0.7475862389 1.8833115996

H48 -5.7947670206 -0.7118277726 0.9177678850

Ru49 -2.8277525828 -0.5147840842 10.6583728710

O50 -3.5787858218 -0.0182013239 8.9419610166

C51 -1.8214652345 -4.5222131892 12.2055815161

C52 -2.3168370164 -3.4378932717 11.4628276502

N53 -2.1735961069 -2.1836042671 11.8789965445

O54 -3.3648934647 -2.3223068784 9.7245585445

C55 -3.0974798760 -3.5114441008 10.1833469899

O56 -3.4583682926 -4.5631479271 9.6758848645

C57 -7.6212461189 -1.1200997892 11.1557109951

C58 -6.7330508797 -1.9822694796 11.8159486730

C59 -5.3830194825 -1.6881093034 11.8468828393

N60 -4.8736868548 -0.5804924500 11.2678799764

C61 -5.7257269275 0.3177865457 10.7336545676

C62 -7.0941781852 0.0813310552 10.6746023742

C63 1.8460342250 -0.0799812682 9.0012277039

C64 1.0594161607 1.0208564031 9.3621301518

C65 -0.2423503081 0.8414819632 9.8026760497

N66 -0.7980265477 -0.3816023468 9.9336311351

C67 -0.0643888377 -1.4546410441 9.5753202276

C68 1.2390844030 -1.3386590125 9.1051340513

H69 -1.9753155846 -5.5187943589 11.8058124896

H70 -7.0851754457 -2.9046692100 12.2671656388

H71 -4.6778798136 -2.3650467889 12.3116824155

H72 -5.2747315720 1.2037770899 10.3021642267

H73 -7.7331997540 0.8169899601 10.1970834952

H74 1.4540198865 2.0300625497 9.2946139662

H75 -0.8773518688 1.6851820955 10.0338440182

H76 -0.5380566446 -2.4241940855 9.6292131791

H77 1.7699250964 -2.2436686446 8.8261169656

C78 -1.0319402940 -2.9590244947 13.8792259039

C79 -1.5554140516 -1.9446118631 13.0564991625

C80 -0.4000997945 -1.2209211357 15.4929941750

C81 -0.4339495064 -2.5398696595 15.1197172738

H82 0.0465882111 -0.9400805179 16.4414811346

C84 -1.5123074799 -0.5896981639 13.4362872980

C85 -0.9546164208 -0.1842576666 14.6617778186

C86 -1.0148967543 1.2028454448 14.9517495359

C87 -1.6043091671 2.0641670524 14.0402790621

C88 -2.1192652768 1.5538973982 12.8343571598

N89 -2.0712862187 0.2622617767 12.5501740833

H90 -0.6056787245 1.5809168572 15.8840715572

H91 -1.6860400687 3.1325523919 14.2103853697

C92 -2.7480830764 2.3560585751 11.7303186330

O93 -3.0091531745 1.6056665140 10.6809006635

O94 -2.9446564511 3.5502815826 11.8240381594

C95 -1.1714466675 -4.2886513301 13.4079622383

H96 -0.7829418247 -5.1156178219 13.9952382011

C97 -9.0549741646 -1.5366654433 10.9356626746

H98 -9.5046278449 -1.7776585998 11.9066067776

H99 -9.6326370928 -0.7047871582 10.5189046737

C100 -9.1906397592 -2.7829123149 10.0227472030

C104 -8.9875369344 -2.5394840526 8.5089069929

C107 3.2843939793 0.0970942668 8.5599390895

H108 3.5916951675 1.1308199822 8.7556096787

H109 3.9111616427 -0.5366821774 9.1998573542

C110 3.0123494407 0.6824508018 6.0141947037

C114 3.6193708872 -0.2539545746 7.0912264166

H107 -10.2023121017 -3.1818274414 10.1503052445

H110 -8.5063368816 -3.5680706891 10.3658924915

H111 -9.2851855624 -3.4571304829 7.9860782589

H112 -9.6821739538 -1.7585352684 8.1789226396

H113 3.3325681024 -1.2920679738 6.8833701285

H114 4.7091730993 -0.2132528698 6.9932785649

H115 3.1828655264 1.7288007697 6.2905540169

H116 3.5528147230 0.5127805270 5.0749693197

**Data file S7**

partial charge parameters

Ru1 0.421462

O2 -0.168006

C3 -0.195456

C4 0.258996

N5 -0.34421

O6 -0.325672

C7 0.511016

O8 -0.484916

C9 0.26842

C10 -0.282688

C11 0.138906

N12 -0.15903

C13 0.15169

C14 -0.276006

C15 0.27902

C16 -0.278694

C17 0.055788

N18 -0.06985

C19 0.10092

C20 -0.238484

H21 0.164468

C22 -0.202936

H23 0.169706

H24 0.05085

H25 0.11528

H26 0.182894

C27 -0.20541

H28 0.172836

H29 0.128592

H30 0.114578

H31 0.138182

C32 0.022096

C33 0.25633

C34 -0.184842

C35 -0.159028

H36 0.17729

H37 0.167316

C38 0.087966

C39 0.101728

C40 -0.050242

C41 -0.188234

C42 0.15142

N43 -0.188554

H44 0.153158

H45 0.181386

C46 0.57608

O47 -0.428434

O48 -0.474244

C49 -0.018242

H50 0.14391

Ru51 0.521758

O52 -0.222498

C53 -0.170236

C54 0.111052

N55 -0.169742

O56 -0.439808

C57 0.595134

O58 -0.470228

C59 0.183162

C60 -0.178224

C61 -0.055374

N62 0.0074

C63 0.075064

C64 -0.185252

C65 0.371096

C66 -0.353074

C67 0.206852

N68 -0.257124

C69 0.225984

C70 -0.352166

H71 0.173516

C72 -0.18061

H73 0.158892

H74 0.151794

H75 0.095526

H76 0.131002

C77 -0.260044

H78 0.178884

H79 0.052786

H80 0.104588

H81 0.20401

C82 0.076878

C83 0.084432

C84 -0.190986

C85 -0.166754

H86 0.178304

H87 0.17056

C88 0.281262

C89 0.060772

C90 -0.044836

C91 -0.195234

C92 0.27805

N93 -0.406944

H94 0.149398

H95 0.164848

C96 0.439114

O97 -0.307786

O98 -0.462866

C99 -0.036478

H100 0.1455

H101 0.08318

H102 0.08551

C103 0.053456

H104 0.105834

H105 0.100102

C106 0.094816

H107 0.05583

H108 0.018108

H109 0.105564

H110 0.082778

H111 0.010564

H112 0.047546

H113 0.079996

H114 0.086226

**Rreferences**

[1] Bard, A. J., *Electrochemical methods : fundamentals and applications* Wiley: New York, 1980.

[2] Huynh, M.; Bediako, D. K.; Nocera, D. G., "A functionally stable manganese oxide oxygen evolution catalyst in acid," *Journal of the American Chemical Society,*  vol. 136, no. 16, pp. 6002-10, **2014**.

[3] Bamford, C. H.; Tipper, C. F. H.; Compton, R. G., *Electrode Kinetics: Principles and Methodology: Principles and Methodology*. Elsevier: 1986.

[4] Chen, Z.; Concepcion, J. J.; Hu, X.; Yang, W.; Hoertz, P. G.; Meyer, T. J., "Concerted O atom–proton transfer in the O—O bond forming step in water oxidation," *Proceedings of the National Academy of Sciences,*  vol. 107, no. 16, pp. 7225, **2010**.

[5] Zhuo, Q.; Zhan, S.; Duan, L.; Liu, C.; Wu, X.; Ahlquist, M. S.; Li, F.; Sun, L., "Tuning the O–O bond formation pathways of molecular water oxidation catalysts on electrode surfaces via second coordination sphere engineering," *Chinese Journal of Catalysis,*  vol. 42, no. 3, pp. 460-469, **2021**.

[6] Odrobina, J.; Scholz, J.; Pannwitz, A.; Francàs, L.; Dechert, S.; Llobet, A.; Jooss, C.; Meyer, F., "Backbone Immobilization of the Bis(bipyridyl)pyrazolate Diruthenium Catalyst for Electrochemical Water Oxidation," *ACS Catalysis,*  vol. 7, no. 3, pp. 2116-2125, **2017**.

[7] Ashford, D. L.; Sherman, B. D.; Binstead, R. A.; Templeton, J. L.; Meyer, T. J., "Electro-Assembly of a Chromophore-Catalyst Bilayer for Water Oxidation and Photocatalytic Water Splitting," *Angewandte Chemie International Edition,*  vol. 54, no. 16, pp. 4778-4781, **2015**.

[8] Li, W.; Li, F.; Yang, H.; Wu, X.; Zhang, P.; Shan, Y.; Sun, L., "A bio-inspired coordination polymer as outstanding water oxidation catalyst via second coordination sphere engineering," *Nature Communications,*  vol. 10, no. 1, pp. 5074, **2019**.

[9] Li, F.; Yang, H.; Zhuo, Q.; Zhou, D.; Wu, X.; Zhang, P.; Yao, Z.; Sun, L., "A Cobalt@Cucurbit[5]uril Complex as a Highly Efficient Supramolecular Catalyst for Electrochemical and Photoelectrochemical Water Splitting," *Angewandte Chemie International Edition,*  vol. 59, no. 59, pp. 2-12, **2020**.

[10] Bard, A., *Standard potentials in aqueous solution*. Routledge: 2017.

[11] Malko, D.; Kucernak, A., "Kinetic isotope effect in the oxygen reduction reaction (ORR) over Fe-N/C catalysts under acidic and alkaline conditions," *Electrochemistry Communications,*  vol. 83, no., pp. 67-71, **2017**.

[12] Bard, A. J.; Faulkner, L. R., *Electrochemical methods: fundamentals and applications*. Wiley New York: 1980; Vol. 2.

[13] Kohen, A.; Limbach, H.-H., *Isotope effects in chemistry and biology*. cRc Press: 2005.

[14] Tong, L.; Duan, L.; Xu, Y.; Privalov, T.; Sun, L., "Structural modifications of mononuclear ruthenium complexes: a combined experimental and theoretical study on the kinetics of ruthenium-catalyzed water oxidation," *Angewandte Chemie, International Edition in English,*  vol. 50, no. 2, pp. 445-9, **2011**.

[15] Zhuo, Q.; Zhan, S.; Duan, L.; Liu, C.; Wu, X.; Ahlquist, M. S. G.; Li, F.; Sun, L., "Tuning the O–O bond formation pathways of molecular water oxidation catalysts on electrode surfaces via second coordination sphere engineering," *Chinese Journal of Catalysis,*  vol. 42, no. 3, pp. 460-469, **2021**.

[16] Duan, L.; Xu, Y.; Gorlov, M.; Tong, L.; Andersson, S.; Sun, L., "Chemical and Photochemical Water Oxidation Catalyzed by Mononuclear Ruthenium Complexes with a Negatively Charged Tridentate Ligand," *Chemistry – A European Journal,*  vol. 16, no. 15, pp. 4659-4668, **2010**.

[17] Rabten, W.; Kärkäs, M. D.; Åkermark, T.; Chen, H.; Liao, R.-Z.; Tinnis, F.; Sun, J.; Siegbahn, P. E. M.; Andersson, P. G.; Åkermark, B., "Catalytic Water Oxidation by a Molecular Ruthenium Complex: Unexpected Generation of a Single-Site Water Oxidation Catalyst," *Inorganic Chemistry,*  vol. 54, no. 10, pp. 4611-4620, **2015**.
